# PSMA-targeted radioligand therapy with beta emitters in prostate cancer: current evidence, emerging indications, combination strategies, and future directions

**DOI:** 10.3389/fmed.2026.1925228

**Published:** 2026-09-25

**Authors:** Hiba Noamen, Adam Bekkhoucha, Alice Bernard Tessier, Khali Trabelsi, Mehdi Borsali, Natacha Naoun, Macarena Rey-Cardenas, Alessia Daverio, Ronan Flippot, Cedric Pobel, Lison Ferreol, Anna Patrikidou, Solenn Barraud, Jacopo Uliano, Alexandre Megherbi, Mario Terlizzi, Laurence Albiges, Pierre Blanchard, Mario Dipalma, Yohann Loriot, Camilo Garcia, Désirée Deandreis

**Affiliations:** 1 Department of Nuclear Medicine, Gustave Roussy, Paris Saclay University, Villejuif, France; 2 Department of Cancer Medicine, Gustave Roussy, Paris Saclay University, Villejuif, France; 3 Radiopharmacy Unit, Pharmacy Department, Gustave Roussy, Paris Saclay University, Villejuif, France; 4 Department of Radiotherapy, Gustave Roussy, Paris Saclay University, Villejuif, France

**Keywords:** beta emitters, prostate cancer, radiopharmaceutical therapies, targeted radioligand therapy (TRT), theranosctics

## Abstract

Prostate-specific membrane antigen (PSMA)-targeted radioligand therapy (RLT) has emerged as a major therapeutic advance in metastatic castration-resistant prostate cancer (mCRPC), The phase III VISION trial demonstrated that [^177^Lu]Lu-PSMA-617 significantly improves overall survival and radiographic progression-free survival (rPFS) establishing PSMA-targeted RLT as a key treatment option in advanced disease. Prostate-specific Membrane Antigen (PSMA) positron emission tomography/computed tomography (PET/CT) plays a central role in patient selection, treatment monitoring, and prognostic stratification, thereby supporting a precision oncology approach. More recently, the clinical development of PSMA-targeted radioligand therapy (RLT) has expanded in several directions, including its use in earlier disease stage, such as metastatic hormone-sensitive prostate cancer (mHSPC), and in the neoadjuvant and oligometastatic settings, as well as its integration into multimodal treatment strategies. These include combination approaches with androgen receptor pathway inhibitors (ARPIs), chemotherapy, immunotherapy, and radiation therapy. This review provides a comprehensive overview of the major developments in the field, including advances in molecular imaging, emerging therapeutic indications, combination approaches, and future directions in PSMA-targeted theranostics according to recent clinical trial data. Collectively, these developments highlight the evolving role of beta-emitting RLT across the prostate cancer disease.

## 1 Introduction

Prostate cancer (PCa) is the second most frequently diagnosed malignancy and the fourth leading cause of cancer death among men worldwide, with an estimated 1.55 million new cases and approximately 420,000 deaths in 2024 according to GLOBOCAN estimates (1, 1a). It remains a leading cause of cancer-related morbidity and mortality, with incidence rates almost three times higher in transitioned than in transitioning countries (34.9 vs. 13.1 per 100,000), whereas mortality rates differ more modestly (7.4 vs. 6.9 per 100,000), reflecting disparities in early detection and access to curative treatment (1a) (1). Although earlier diagnosis and major therapeutic advances have improved clinical outcomes, the management of advanced and recurrent disease continues to represent a significant clinical challenge. In this context, radioligand therapy (RLT) has emerged as an effective and rapidly expanding systemic treatment modality.

RLT involves the systemic administration of radiolabelled compounds designed to selectively bind tumour-associated targets, thereby delivering cytotoxic radiation directly to cancer cells while limiting exposure to surrounding healthy tissues.

A shift arose by targeting prostate-specific membrane antigen (PSMA), a transmembrane glycoprotein that is highly overexpressed in most prostate cancer cells and metastatic lesions, making it an attractive target for theranostic applications by the beta emitter radioligand [^177^Lu]Lu-PSMA (2). This approach can effectively target all prostate cancer lesions independently from their origin including nodal, bone and visceral metastases. Beta particles have a long tissue penetration range (0.5–2 mm) enabling a crossfire effect that facilitates irradiation of neighbouring tumour cells.

The clinical development of PSMA-targeted RLT was established by the phase III VISION trial, which demonstrated the efficacy and safety of [^177^Lu]Lu- vipivotide tetraxetan (PSMA-617) in patients with PSMA-positive mCRPC. In this multinational randomised study involving 831 patients, the addition of [^177^Lu]Lu-PSMA-617 to standard of care (SOC) significantly improved radiographic progression-free survival (rPFS) (8.7 vs. 3.4 months) and overall survival (OS) (15.3 vs. 11.3 months), while also delaying the time to first symptomatic skeletal event (11.5 vs. 6.8 months). These findings supported the regulatory approval of [^177^Lu]Lu-PSMA-617 (Pluvicto®) in post ARPI and post-taxane setting in mCRPC. The VISION trial also established the recommended treatment regimen consisting of four to six cycles of 7.4 GBq administered at six-week intervals (3).

Patient selection is a critical determinant of treatment efficacy in PSMA-targeted RLT. Eligibility depends on the *in vivo* demonstration of sufficient target expression throughout the overall tumour burden, as heterogeneous or low PSMA expression may reduce the likelihood of clinical benefit. Assessment of PSMA expression is achieved using PSMA positron emission tomography/computed tomography (PSMA PET/CT), which enables whole-body evaluation of disease distribution and target expression. Beyond visual image interpretation, novel imaging biomarkers and standardised assessment strategies have been developed to improve patient stratification, refine prognostic and predictive evaluation, and facilitate treatment response assessment.

From a therapeutic perspective, ongoing and forthcoming studies are expected to further expand the role of PSMA-targeted RLT across the prostate cancer disease continuum. One of the most promising developments is its evaluation in earlier disease stages, including metastatic hormone-sensitive prostate cancer (mHSPC), as well as in high-risk localised disease within neoadjuvant and adjuvant treatment strategies.

Another major area of investigation is the combination of beta-emitting RLT with other systemic therapies, including androgen receptor pathway inhibitors (ARPIs), chemotherapy, targeted agents such as poly(ADP-ribose) polymerase (PARP) inhibitors, immunotherapy and external beam radiation. Emerging clinical evidence suggests that these combinations may enhance therapeutic efficacy through synergistic mechanisms, including increased PSMA expression, radiosensitisation, modulation of the tumour microenvironment, and the targeting of treatment-resistant tumour subclones.

## 2 The importance of molecular imaging

Patient selection for PSMA-targeted RLT relies critically on PSMA PET/CT, which enables whole-body assessment of tumour PSMA expression and represents a cornerstone of the theranostic paradigm in prostate cancer. Its highly sensitive detection of primary and metastatic lesions allows precise characterisation of disease extent and intrapatient heterogeneity, identifying patients with sufficient PSMA expression for effective RLT targeting (2) while excluding those with low or heterogeneous expression unlikely to benefit.

Several PSMA-targeting PET tracers are widely used, including [^68^Ga]Ga-PSMA-11, [^18^F]F-PSMA-1007, and [^18^F]F-DCFPyL, though their regulatory approval status varies across jurisdictions. Notably, [^68^Ga]Ga-PSMA-11 was used for patient selection in the VISION trial (3), whereas other agents have been approved for diagnostic indications without specific validation within the original phase III RLT framework. Despite recognised differences in biodistribution and quantitative uptake, their impact on clinical patient selection remains to be fully established, with real-world practice often driven by local availability and regulatory constraints. Fluorine-18-based radiopharmaceuticals such as piflufolastat F-18 and flotufolastat F-18 have been developed for prostate cancer imaging, with recent in silico investigations exploring their interactions with biological targets relevant to prostate cancer diagnosis, further expanding the diagnostic armamentarium in PSMA-based theranostics (4).

Beyond its diagnostic role, PSMA PET is increasingly recognised as a source of quantitative imaging biomarkers (59). Advances in PET technology and image analysis now allow comprehensive assessment of tumour burden through volumetric and intensity-based parameters, improving patient stratification, prognostic evaluation, and response assessment.

### 2.1 Patient selection and criteria for response assessment at PSMA PET

The concept of PSMA PET positivity is fundamental to patient selection for PSMA-targeted RLT and many attempts have been done to standardize interpretation criteria The PROMISE (Prostate Cancer Molecular Imaging Standardised Evaluation) framework, defines a PSMA-positive lesion in case of tracer uptake exceeding blood-pool activity and visually greater than the surrounding background (5).

The Version 2 of PROMISE criteria also incorporates the molecular imaging TNM (miTNM) classification, the reference organ thresholds (liver and parotid glands), and a four-point grading system. A score of ≥2, corresponding to uptake greater than liver background activity, is generally considered sufficient for PSMA-targeted RLT. Reporting both the highest and lowest lesion scores additionally provides insight into intrapatient tumour heterogeneity, which may be particularly relevant in advanced disease (6).

Version 2.0 also introduced the PSMA-RADS-5T category to improve the characterisation of treated lesions, facilitate standardised interpretation during follow-up, and support clinical decision-making (7, 8).

In metastatic castration-resistant prostate cancer (mCRPC), treatment response has traditionally been assessed using conventional imaging modalities, including computed tomography and bone scintigraphy, according to RECIST 1.1 (9) and PCWG3 criteria (10). However, these approaches remain suboptimal for evaluating targeted therapies such as PSMA-RLT because they do not capture the functional and molecular changes detectable on PSMA PET.

To address these limitations, PSMA PET-specific response criteria have been developed. The Response Evaluation Criteria in PSMA PET/CT (RECIP 1.0) framework integrates changes in whole-body PSMA tumour volume with the occurrence of new lesions. Progressive disease (PD) is defined by the appearance of new PSMA-avid lesions together with a >20% increase in whole-body tumour volume. Partial response (PR) corresponds to a reduction of at least 30% in tumour volume without new lesions, whereas complete response (CR) is characterised by the disappearance of all PSMA-avid disease. Stable disease (SD) is assigned when neither PR nor PD criteria are met (11).

A notable strength of RECIP 1.0 is the incorporation of qualitative visual assessment by experienced readers, facilitating implementation in routine clinical practice without the need for dedicated segmentation software.

In a multicentre retrospective study involving 124 patients with mCRPC treated with [^177^Lu]Lu-PSMA, RECIP 1.0 demonstrated superior prognostic performance compared with RECIST 1.1, adapted PCWG3 criteria, and other PET-based response frameworks. RECIP showed the highest predictive value for overall survival (hazard ratio 4.33), together with excellent interobserver agreement (*κ* = 0.83), highlighting its potential clinical utility (11).

These findings were subsequently reinforced by a multicentre analysis demonstrating high concordance (95%) between qualitative and quantitative RECIP assessments (*κ* = 0.89), together with strong inter-reader agreement. RECIP-defined progressive disease was significantly associated with shorter overall survival (HR = 2.6; *p* < 0.001), further supporting its prognostic relevance and practical applicability (12).

Despite these encouraging results, RECIP-based frameworks still require prospective validation before they can be considered fully established in routine clinical practice and incorporated into regulatory clinical trial endpoints.

In parallel, the updated recommendations from the Prostate Cancer Working Group 4 (PCWG4) are expected to further refine clinical trial design and outcome assessment by incorporating molecular imaging, particularly PSMA PET, into disease classification and response evaluation frameworks. Importantly, these recommendations acknowledge disease states that may only be detectable by molecular imaging, including oligometastatic and micrometastatic disease, thereby better reflecting the contemporary landscape of prostate cancer management (13).

The Standardised PSMA PET/CT Analysis and Reporting Consensus (SPARC) recommendations have also been developed to harmonise image interpretation, reporting practices, and clinical communication, promoting greater consistency in patient selection and treatment assessment for PSMA-targeted RLT (14). These recommendations emphasise the importance of standardised acquisition protocols, quantitative PET metrics, and structured reporting criteria to improve comparability across clinical trials and real-world practice. In particular, SPARC highlights the value of whole-body tumour burden metrics and the integration of molecular imaging with clinical and biological parameters to improve patient stratification and prognostic assessment.

Nevertheless, despite increasing standardisation efforts, variability in acquisition protocols, interpretation thresholds, and tracer availability continues to affect reproducibility across institutions.

#### 2.1.1 False-positive PSMA PET findings

Although PSMA PET/CT demonstrates high sensitivity for prostate cancer detection, false-positive findings may occur and influence clinical decision-making. Physiological uptake in benign prostatic tissue, ganglia, and certain normal organs may mimic pathological uptake. More importantly, non-malignant conditions including Paget disease, fibrous dysplasia, haemangiomas, and inflammatory or infectious processes may demonstrate PSMA avidity, potentially leading to overstaging or inappropriate treatment selection. Salivary and lacrimal gland uptake, while predictable, may obscure adjacent nodal disease. Urinary excretion of certain tracers, particularly [^68^Ga]Ga-PSMA-11 and [^18^F]F-DCFPyL, may obscure pelvic lymph node assessment. Standardised interpretation frameworks such as PROMISE and PSMA-RADS have been developed to mitigate these challenges, yet interobserver variability and tracer-specific differences in biodistribution continue to affect diagnostic accuracy.

### 2.2 The role of FDG PET/CT

In selected patients [^18^F]F-FDG PET provides complementary information to PSMA PET by identifying biologically aggressive and metabolically active tumour clones. This dual-tracer approach is particularly valuable for detecting PSMA-negative/FDG-positive lesions, which have consistently been associated with treatment resistance and adverse clinical outcomes (15–17).

In the phase II TheraP trial, baseline FDG PET identified patients with a high metabolic tumour volume (MTV ≥ 200 mL), who exhibited lower PSA response rates and an increased risk of both disease progression (HR = 1.89) and death (HR = 2.04). Randomised comparison demonstrated superior progression-free survival with [^177^Lu]Lu-PSMA-617 compared with cabazitaxel (HR = 0.63, 95% CI = 0.46–0.86; *p* = 0.0050), whereas overall survival was similar between treatment groups (median 16.4 vs. 19.4 months; HR 0.97; *p* = 0.99). Patients excluded because of FDG/PSMA mismatch experienced markedly poorer outcomes, with a restricted mean survival time of 11.0 months (95% CI = 9.0–13.1), underscoring the adverse prognostic significance of discordant disease (18).

Beyond qualitative assessment, quantitative parameters derived from PSMA PET have emerged as potential biomarkers of treatment response. Among these, whole-body SUVmean has attracted particular interest as a surrogate of global target expression. Higher SUVmean values have been associated with improved biochemical response and prolonged survival following PSMA-targeted RLT, suggesting that quantitative PET metrics may further refine patient selection beyond conventional visual assessment (17).

Taken together, FDG discordance and quantitative PSMA PET biomarkers appear to play complementary roles. Whereas FDG-positive/PSMA-negative disease primarily identifies patients less likely to benefit from PSMA-targeted RLT, quantitative measures of PSMA expression may help identify those most likely to derive substantial therapeutic benefit.

## 3 RLT in different prostate cancer disease settings

### 3.1 RLT in the the metastatic castration-resistant prostate cancer setting

Beyond VISION several clinical trials have evaluated the efficacy of [^177^Lu]Lu-labelled PSMA-targeted radioligand therapy in metastatic castration-resistant prostate cancer (mCRPC), particularly before exposure to taxane chemotherapy.

The PSMAfore trial (NCT04689828) a multicentre, open-label, randomised phase III demonstrated a significant improvement in radiographic progression-free survival (rPFS) (median 12.0 vs. 5.6 months; HR = 0.41, 95% CI = 0.29–0.56; *p* < 0.0001) and objective response rate (50.7% vs. 14.9%) with [^177^Lu]Lu-PSMA-617 compared with an androgen receptor pathway inhibitor (ARPI) switch in taxane-naïve patients with mCRPC. These findings support the use of [^177^Lu]Lu-PSMA-617 earlier in the disease course and contributed to its approval by the U.S. Food and Drug Administration in this setting (19).

The SPLASH trial (NCT04647526) is another multicentre, open-label, randomised phase III study evaluating [^177^Lu]Lu-PSMA-I&T (PNT2002) in patients with PSMA-positive mCRPC in the same setting (20). In the primary analysis, [^177^Lu]Lu-PSMA-I&T significantly improved rPFS compared with ARPI switch (median 9.5 vs. 6.0 months; HR = 0.71, 95% CI = 0.55–0.92; *p* = 0.0088), while also achieving higher objective response rates (38.1% vs. 12.0%; *p* = 0.0021) and PSA50 response rates (35.7% vs. 14.6%). In both trials PSMA treatment was generally well tolerated, with grade ≥3 treatment-related adverse events reported in 9.7% of patients receiving [^177^Lu]Lu-PSMA-I&T (20).

The design of both PSMAfore and SPLASH raises significant methodological concerns. First, both trials compare PSMA-targeted RLT against an ARPI switch—a comparator with limited clinical activity and questionable validity as a modern standard of care, given that contemporary guidelines favour taxane-based chemotherapy following progression on initial ARPI therapy. Second, both studies employed permissive crossover, confounding overall survival analyses and necessitating non-pre-specified sensitivity analyses that limit interpretability. Third, inclusion criteria selected patients eligible for ARPI switching—implicitly enriching for slower disease progression—potentially favouring RLT outcomes. Fourth, differences in dosing and scheduling between the two trials (PSMAfore: 7.4 GBq every 6 weeks; SPLASH: 6.0 GBq every 8 weeks for PNT2002) preclude direct cross-trial comparison and raise questions about optimal therapeutic regimens. Collectively, these limitations—non-standard comparators, crossover confounding, selection bias toward indolent phenotypes, and protocol heterogeneity—undermine the ability of either trial to definitively establish the therapeutic value of PSMA-targeted RLT in this setting.

The ECLIPSE trial (NCT05204927) is a pivotal, multicentre, open-label, phase III randomised study evaluating [^177^Lu]Lu-PSMA-I&T vs. ARPI switch (abiraterone acetate or enzalutamide) in docetaxel-naïve patients with mCRPC (21) with the same design as for the PSMA Fore and SPLASH.

Collectively, these studies indicate that PSMA-targeted RLT is progressively moving from a late-line treatment option towards an earlier therapeutic intervention within the mCRPC disease continuum. Beyond demonstrating efficacy, they are helping to define the optimal timing of treatment, refine patient selection, and clarify whether the clinical benefits observed with [^177^Lu]Lu-PSMA-617 can be generalised across different PSMA-targeting ligands (Table 1).

**Table 1:** Comparison of PSMAfore trial, SPLASH trial and ECLIPSE trials in the metastatic castration-resistant prostate cancer in pre-docetaxel setting.

Characteristic	PSMA Fore [^177^Lu] Lu-PSMA-617 arm vs. switch ARPI		SPLASH [^177^Lu] Lu-PSMA-I&T arm vs. Alternate ARPI arm		ECLIPSE (CURLu177PSMA0001) [^177^Lu] Lu-PSMA-I&T arm vs. Alternate ARPI arm	
Randomization	(1:1)		(2:1)		(2:1)	
Treatment regimen	Up to 6 × 7.4 GBq every 6 weeks	Abiraterone Acetate with Prednisone or Enzalutamide	4 × 6.8 GBq every 8 weeks	Abiraterone Acetate with Prednisone or Enzalutamide	Up to 6 × 7.4 GBq every 6 weeks	Abiraterone Acetate with Prednisone or Enzalutamide
Number of patients	115	168	276	136	267	133
Median Follow up	12.7 m	13 m	11 m	13 m	NR	NR
rPFS	11.6 m [HR = 0.49 (0.39–0.61)]	6 m	9.5 m [HR = 0.71 (0.55–0.92)]	6 m	NR	NR
ORR	50.7%	14.9%	38.1%	12%	NR	NR
PSA50 response	57.6%	20.4%	35.7%	14.6%	NR	NR
Adverse events >Grade 3	36%	48%	30%	36%	NR	NR
Crossover	No	84.2%	No	84.6%	No	Allowed to receive [^177^Lu] Lu-PSMA-I&T after progression

ARPI, androgen receptor pathway inhibitor; CI, confidence interval; HR, hazard ratio; mCRPC, metastatic castration-resistant prostate cancer; NR, not reported; ORR, objective response rate; PSA50 response, ≥50% decline in prostate-specific antigen from baseline; RLT, radioligand therapy; rPFS, radiographic progression-free survival.

Beyond small-molecule PSMA ligands, alternative radiopharmaceutical approaches are also being explored. Antibody-based agents possess pharmacokinetic properties that differ substantially from those of conventional small-molecule PSMA ligands, including prolonged circulation time, delayed tumour uptake, and extended tumour retention.

[^177^Lu]Lu-rosopatamab tetraxetan (TLX591) is a PSMA-targeted radioimmunoconjugate consisting of a monoclonal antibody linked to [^177^Lu] via a DOTA chelator. The compound is characterised by hepatic clearance, prolonged tumour retention and internalisation, and a fractionated dosing regimen consisting of two intravenous administrations delivered 14 days apart.

The ProstACT GLOBAL trial is an ongoing multinational, multicentre, prospective, randomised, open-label phase III study evaluating [^177^Lu]Lu-rosopatamab tetraxetan in patients with PSMA-positive mCRPC whose disease has progressed following one ARPI (22). Patients are randomised in a 2:1 ratio to receive [^177^Lu]Lu-rosopatamab tetraxetan plus standard of care or standard of care alone. Radiographic progression-free survival serves as the primary endpoint, while overall survival and objective response rate are included among the key secondary endpoints (22).

The development of TLX591 reflects a broader trend within prostate cancer theranostics: the exploration of alternative targeting platforms designed to optimise tumour retention, radiation delivery, and biological efficacy. Whether antibody-based approaches can offer clinically meaningful advantages over established small-molecule ligands remains an important unanswered question that will require prospective comparative evaluation.

### 3.2 The hormone-sensitive setting

The hormone-sensitive setting represents a major area of development for PSMA-targeted RLT, with the objective of improving disease control earlier in the natural history of prostate cancer.

The PSMAddition trial is a phase III randomised study evaluating [^177^Lu]Lu-PSMA-617 in combination with androgen deprivation therapy (ADT) and an androgen receptor pathway inhibitor (ARPI) in patients with newly diagnosed metastatic hormone-sensitive prostate cancer (mHSPC) (23). Interim results demonstrated a significant improvement in radiographic progression-free survival (rPFS) (HR = 0.72, 95% CI = 0.58–0.90; *p* = 0.002), corresponding to a 28% reduction in the risk of radiographic progression or death, with median rPFS not reached in either treatment arm after a median follow-up of 23.6 months. The benefit was consistent across clinically relevant subgroups, including patients with high- and low-volume disease as well as those with *de novo* and recurrent metastatic disease. Objective response rates according to RECIST 1.1 were 85.3% and 80.8% in the combination and control arms, respectively. Overall survival data remain immature (HR = 0.84, 95% CI = 0.63–1.13; *p* = 0.125), and longer follow-up will be required to determine the impact of this strategy on long-term clinical outcomes (24).

The ongoing PEACE-6 Poor Responders (EU CT n°: **2022-502408-57-00**) trial is evaluating the addition of PSMA-targeted RLT in patients with mHSPC who exhibit a suboptimal PSA response (defined as PSA ≥ 0.2 ng/mL) following initial systemic treatment (25). This biomarker-driven approach is particularly appealing, as it seeks to identify patients with an incomplete biochemical response who may derive the greatest benefit from treatment intensification before the development of castration-resistant disease.

Similarly, STAMPEDE-2 (NCT06320067) is a multi-arm, multi-stage phase III platform trial investigating [^177^Lu]Lu-PSMA-617 in combination with standard-of-care ADT and androgen receptor signalling inhibition in patients with newly diagnosed mHSPC (26). In contrast to the sequential strategies explored in the mCRPC setting, STAMPEDE-2 employs an accelerated administration schedule, consisting of 7.4 GBq on days 1 and 8 followed by three additional cycles at six-week intervals. This design aims to exploit the transient upregulation of PSMA expression induced by the initiation of ADT and ARPI therapy. Patients are randomised 1:1 to receive standard-of-care alone or in combination with [^177^Lu]Lu-PSMA-617, with overall survival as the primary endpoint. By introducing RLT at the earliest stage of metastatic disease, this trial represents a paradigm shift from the treatment sequencing strategies evaluated in PSMAfore and SPLASH. In addition, the platform design allows the incorporation of future investigational arms, providing considerable flexibility for therapeutic innovation (26).

Further support for the use of PSMA-targeted RLT in earlier disease stages comes from the phase II UpFrontPSMA trial. In this study, two cycles of [^177^Lu]Lu-PSMA-617 (7.5 GBq every six weeks) followed by six cycles of docetaxel (75 mg/m^2^ every three weeks) demonstrated superior antitumour activity compared with docetaxel alone in patients with *de novo* high-volume mHSPC. Undetectable PSA levels at 48 weeks were achieved in 41% vs. 16% of patients (OR = 3.88; *p* = 0.002), while PSA progression-free survival was significantly prolonged (median 30 vs. 21 months; HR = 0.60, 95% CI = 0.37–0.98), supporting the potential role of PSMA-targeted RLT in treatment intensification strategies for hormone-sensitive disease (27).

Collectively, these studies suggest that PSMA-targeted RLT may become an important component of treatment intensification in mHSPC. However, its definitive place in clinical practice will depend on the maturation of phase III data, particularly overall survival outcomes and long-term safety. In addition, several questions remain unresolved, including the optimal integration of RLT with contemporary systemic therapies, the appropriate number of treatment cycles, and whether treatment strategies should be adapted according to disease volume and tumour burden (Table 2).

**Table 2:** PSMA-targeted radioligand therapy trials in hormone-sensitive settings.

Trial	Phase/design	Population	Number of patients (included/ planned)	Randomization	RLT used	Intervention arm	Control arm	RLT schedule and injected activity	Primary endpoint(s)
PSMAddition	International, open-label, prospective, randomized phase III trial	Treatment-naïve mHSPC	1,126 included	1:1 randomization	[177Lu] Lu-PSMA-617	Standard of care (ADT + ARPI) + [177Lu] Lu-PSMA-617	Standard of care (ADT + ARPI); (crossover to [177Lu] Lu-PSMA-617 allowed)	[177Lu] Lu-PSMA-617, 7.4 GBq, every 6 weeks for 6 cycles	rPFS
PEACE-6 Poor Responders	International, multicentre, open label, controlled, randomized phase III trial	*De novo* mHSPC patients without disease progression but with PSA ≥ 0.2 ng/mL at 6–8 months after treatment initiation (ADT, ARPI and a maximum of 6 cycles of docetaxel)	500 planned	1:1 randomization	[177Lu] Lu-PSMA-617	Ongoing Standard of care (ADT + ARPI) + [177Lu] Lu-PSMA-617	Ongoing Standard of care (ADT + ARPI)	[177Lu] Lu-PSMA-617, 7.4 GBq, every 6 weeks for 4 planned cycles	OS; rPFS
STAMPEDE-2	Randomized, open-label, multi-arm, multi-stage phase III trial	Patients with metastatic prostate cancer responsive to hormone therapy and initiating ADT and ineligible for SABR	1,756 planned	1:1 randomization	[177Lu] Lu-PSMA-617	Standard of care + [177Lu] Lu-PSMA-617	Standard of care alone	[177Lu] Lu-PSMA-617, 7.4 GBq on days 1 and 8, repeated every 6 weeks for 3 cycles	OS
UpFrontPSMA	Multicentre, open-label, randomized phase II trial	Newly diagnosed high-volume mHSPC/mHNPC receiving continuous ADT	130 included	1:1 randomization	[177Lu] Lu-PSMA-617	Sequential [177Lu] Lu-PSMA-617 followed by docetaxel + ADT	Docetaxel + ADT	[177Lu] Lu-PSMA-617, 7.5 GBq, every 6 weeks, up to 2 cycles	Undetecta ble PSA at 48 weeks
BULLSEYE	Open-label, randomized phase II trial	oligometastatic HSPC with ≤5 metastatic lesions before development of hormone-resistant disease	58 planned (43 included)	1:1 randomization	[177Lu] Lu-PSMA-617	[177Lu] Lu-PSMA-617	deferred ADT [crossover to (177Lu) Lu-PSMA-617 permitted]	[177Lu] Lu-PSMA-617, 7.4 GBq, every 6 weeks, up to 2 cycles, with optional additional 2 cycles	Disease progressio n within 6 months
LUNAR	Open-label, randomized, controlled phase II trial	oligorecurrent hormone-sensitive prostate cancer eligible for metastasis-directed SBRT	100 planned	Randomized 1:1	[177Lu] Lu-PNT2002	Neoadjuvant [177Lu] Lu-PNT2002	SBRT alone	[177Lu] Lu-PNT2002, 6.8 GBq per cycle, up to 2 cycles	PFS (based on PSMA PET/CT)

ADT, androgen deprivation therapy; ARPI, androgen receptor pathway inhibitor; GBq, gigabecquerel; HSPC, hormone-sensitive prostate cancer; mHNPC, metastatic hormone-naïve prostate cancer; mHSPC, metastatic hormone-sensitive prostate cancer; OS, overall survival; PET/CT, positron emission tomography/computed tomography; PFS, progression-free survival; PSA, prostate-specific antigen; PSMA, prostate-specific membrane antigen; RLT, radioligand therapy; rPFS, radiographic progression-free survival; SABR, stereotactic ablative body radiotherapy; SBRT, stereotactic body radiotherapy; SoC, standard of care; [177Lu], lutetium-177.

### 3.3 The neoadjuvant setting

The neoadjuvant application of PSMA-targeted radioligand therapy represents an emerging strategy aimed at improving tumour control before definitive local treatment. To date, most available evidence derives from early-phase studies designed primarily to assess feasibility, dosimetry, safety, and pathological response rather than long-term oncological outcomes.

The LuTectomy trial is a phase I/II study evaluating neoadjuvant [^177^Lu]Lu-PSMA-617 in patients with high-risk localised prostate cancer (28). The study demonstrated that administration of one or two cycles of [^177^Lu]Lu-PSMA-617 (5 GBq per cycle, 6 weeks apart) before radical prostatectomy is feasible, well tolerated, and does not compromise surgical safety, while delivering therapeutically relevant radiation doses to the prostate and involved lymph nodes. Dosimetric analyses reported a median absorbed dose of 19.6 Gy (IQR = 11.3–48.4) to the prostate and a median highest tumour absorbed dose of 35.5 Gy (IQR = 19.5–50.1), whereas involved pelvic lymph nodes received a median absorbed dose of 37.9 Gy. Histopathological evidence of treatment effect was observed in approximately 80% of prostatectomy specimens, and 45% of patients achieved a PSA decline exceeding 50%. No complete pathological responses were observed following a single treatment cycle. Although these findings provide proof of concept for neoadjuvant PSMA-targeted RLT, the modest pathological response rates and absence of long-term oncological endpoints preclude definitive conclusions regarding clinical benefit.

Building on these observations, the German NEPI trial (NCT06388369) is investigating a phase I/II strategy combining [^177^Lu]Lu-PSMA-617 with ipilimumab in patients with very high-risk prostate cancer before surgery (29). The rationale is based on the hypothesis that radiation-induced immunogenic cell death may enhance the efficacy of immune checkpoint blockade and increase pathological response rates. However, given its early-phase design, the clinical relevance of this intensified approach remains uncertain.

A prospective phase II study conducted at the University of Vienna (NCT06259123) is evaluating neoadjuvant [^177^Lu]Lu-PSMA-I&T in patients with *de novo* oligometastatic prostate cancer, with endpoints including PSA response, imaging findings, and histopathological outcomes following radical prostatectomy (30). Unlike LuTectomy, which focuses on high-risk localised disease, this study addresses a different clinical question: whether neoadjuvant radioligand therapy can achieve meaningful cytoreduction before surgery in treatment-naïve oligometastatic patients. In this setting, pathological response may represent a valuable translational marker of biological activity.

The PROQURE-I trial (NCT05162573) is a multicentre phase I study investigating the addition of a single cycle of [^177^Lu]Lu-PSMA-617 administered concurrently with external beam radiotherapy (EBRT) to the prostate and pelvic lymph nodes (35 fractions over seven weeks) in combination with androgen deprivation therapy in patients with PSMA-positive N1M0 disease (31). By integrating systemic radioligand therapy into a curative-intent locoregional treatment programme, the study explores a novel multimodal strategy, although its ultimate clinical significance remains to be established. More recently, several additional early-phase studies have broadened the scope of neoadjuvant PSMA-targeted RLT. The STARLiT trial (NCT06574880) is a single-arm phase I/II study evaluating [^177^Lu]Lu-PSMA-617 combined with stereotactic body radiotherapy (SBRT) to the prostate and pelvic lymph nodes as an androgen deprivation therapy-free strategy in locally advanced prostate cancer (32). Similarly, the PRELUDE trial (NCT06798558) is a prospective single-arm phase II study investigating two cycles of [^177^Lu]Lu-PSMA-617 (7.4 GBq every six weeks) before radical prostatectomy in patients with high-risk localised prostate cancer, with pathological downstaging as the primary endpoint (33). The LUPUS study explores pelvic intra-arterial administration of [^177^Lu]Lu-PSMA-617 (34), an innovative approach intended to enhance tumour targeting while potentially reducing systemic exposure. Preliminary findings suggest that intra-arterial delivery is feasible and associated with favourable tumour targeting and dosimetric characteristics. Nevertheless, long-term efficacy and safety data are not yet available.

In the neoadjuvant setting the oligometastatic disease represent another challenge.

BULLSEYE is an open-label, randomised, phase II trial evaluating [^177^Lu]Lu-PSMA-617 monotherapy in men with PSMA-expressing oligometastatic hormone-sensitive prostate cancer (HSPC) (35). Eligible participants had biochemically recurrent disease after radical prostatectomy or radiotherapy, ECOG performance status 0–1, a PSA doubling time of less than 6 months, and PSA above 1.0 µg/L and less than 5 lesions on PET PSMA. Fifty-eight patients were randomised 1:1 to 2 (+2) cycles of 7.4 GBq [^177^Lu]Lu-PSMA-617 vs. standard of care (watchful waiting with deferred ADT), with control-arm patients permitted to cross over to [^177^Lu]Lu-PSMA-617 upon reaching the primary endpoint. The primary endpoint was disease progression, defined as a 100% increase in PSA or radiographic/clinical progression. Patients in the intervention group received a median of four cycles, and at a median follow-up of 27 months, the trial met its endpoint decisively: during the first 30 weeks, progression occurred in 2 of 29 (7%) intervention patients vs. 27 of 29 (93%) control patients (*p* < 0.0001), and per-protocol median progression-free survival was 25 months in the intervention arm vs. 5 months in the control arm (HR = 0.07; 95% CI = 0.03–0.17; *p* < 0.0001). A complete remission was seen in 21% (6/29) of treated patients, and the regimen was well tolerated: the most common treatment-related events were grade 1 dry mouth, fatigue, nausea, and transient bone marrow toxicity, with grade ≥2 events seldom observed. The authors concluded that [^177^Lu]Lu-PSMA-617 could serve as a metastasis-directed option to delay ADT in this population.

The LUNAR trial is single-centre, open-label, randomised, controlled phase II study conducted at UCLA that tested whether adding neoadjuvant PSMA-targeted radioligand therapy to metastasis-directed stereotactic body radiotherapy (SBRT) improves outcomes in men with oligo recurrent hormone-sensitive prostate cancer (orHSPC) (36, 37). Eligible participants had one to five lesions identified on PSMA PET/CT, and after stratification by stage (N1/M1a vs. M1b) and lesion count (1 vs. 2–3 vs. 4–5), patients were randomly assigned 1:1 to receive SBRT to all lesions or two cycles of [^177^Lu]Lu-PNT2002 followed by SBRT to all lesions. 42 patients received SBRT alone and 45 received [^177^Lu]Lu-PSMA treatment plus SBRT; at a median follow-up of about 22 months, median progression-free survival was 7.4 months with SBRT alone vs. 17.6 months with the combination (HR = 0.37; 95% CI = 0.22–0.61; *P* < .0001). Time to initiation of hormonal therapy was also significantly prolonged in the combination arm (HR = 0.40; 95% CI = 0.22–0.71; *P* = .0014). The benefit was achieved without a significant increase in toxicity, with grade ≥3 lymphopenia seen in 7% of the combination arm vs. 5% with SBRT alone and no other grade ≥3 treatment-attributable toxicities. That neoadjuvant [^177^Lu]Lu-PNT2002 added to SBRT seem to double PFS in this population, also in patients with no evidence of disease on PET PSMA supporting the adjuvant scope of [^177^Lu]Lu-PSMA RLT.

Taken together, these studies indicate that neoadjuvant PSMA-targeted RLT is technically feasible and capable of producing measurable biological and pathological effects and preliminary evidence on impact on patient outcome (Table 3).

**Table 3:** PSMA-targeted radioligand therapy trials in localized, locally advanced, oligometastatic and perioperative prostate cancer settings.

Trial/study	Disease setting	Phase/design	Population	Number of patients	Randomization/design structure	RLT used	Treatment strategy	RLT schedule	Primary endpoint(s)
LuTectomy	High-risk localized prostate cancer	Single-centre, single-arm phase I/II study	Men with high-risk localized prostate cancer, and scheduled for robotic radical prostatectomy	20 included	Single arm; two sequential cohorts	[^177^Lu] Lu-PSMA-617	Neoadjuvant RLT before robotic radical prostatectomy	1 or 2 cycles; 5 GBq per cycle; surgery 6 weeks later	Radiation absorbed dose
NEPI	Very high-risk prostate cancer eligible for radical prostatectomy	Randomized, open-label phase I/II study	Very high-risk prostate cancer candidates for radical prostatectomy;	58 planned	Safety run-in followed by 1:1 randomization	[^177^Lu] Lu-PSMA-617	Neoadjuvant [^177^Lu] Lu-PSMA-617 + ADT with or without ipilimumab before radical prostatectomy	2 cycles of 7.4 GBq	Feasibility to perform prostatectomy on time, pathological complete response
University of Vienna study	Oligometastatic prostate cancer planned for radical prostatectomy	Prospective, single-centre phase II pilot study	Oligometastatic primary prostate cancer and planned for radical prostatectomy	10 planned	Single-arm pilot study	[^177^Lu] Lu-PSMA-ICT	Neoadjuvant PSMA-RLT before radical prostatectomy	2 cycles of 5 GBq at 6-week intervals before radical prostatectomy	Safety and tolerability
PROQURE-I	Node-positive non-metastatic prostate cancer	Multicentre prospective phase I dose-escalation study	Treatment-naïve PSMA-positive N1M0 prostate cancer treated with curative intent	Up to 18 planned	Bayesian Optimal Interval dose-escalation design	[^177^Lu] Lu-PSMA-617	Concurrent [^177^Lu] Lu-PSMA-617 with standard EBRT and long-term ADT	One cycle administered during week 2 of EBRT; dose levels of 3, 6, or 9 GBq	maximum tolerated dose (MTD)
STARLiT	Locally advanced prostate cancer	Single-arm, open-label phase I/II trial	PSMA PET/CT-avid locally advanced prostate cancer with pelvic/regional nodal disease or high-risk localized features	45 planned	Single arm; phase I dose escalation followed by phase II expansion	[^177^Lu] Lu-PSMA-617	[^177^Lu] Lu-PSMA-617 combined with prostate and nodal SBRT	Initial 7.4 GBq cycle followed by 5-fraction SBRT; up to two additional 7.4 GBq cycles at 6-week intervals	Phase I: maximum tolerated dose; Phase II: 3-year ADT-free survival
PRELUDE	High-risk localized prostate cancer	Prospective, single-arm phase II study	Men with high-risk localized prostate adenocarcinoma eligible for radical prostatectomy	54 planned	Single arm	[^177^Lu] Lu-PSMA-617	Neoadjuvant [^177^Lu] Lu-PSMA-617 before radical prostatectomy	2 cycles of 7.4 GBq every 6 weeks before surgery	Pathological down-staging
NeoPSMA	high-risk localized prostate cancer	Open-label, multicentre phase I/II study	high-risk localized prostate cancer before radical prostatectomy and pelvic lymph node dissection	228 planned	Open-label, multicentre phase I/II	[177Lu] Lu-PSMA-R2 and [^225^Ac] Ac-PSMA-R2	Neoadjuvant RLT before radical prostatectomy and pelvic lymph node dissection	a single intravenous dose Dose	DLTs, BFS
LUPUS	High-risk localized prostate cancer	Phase I/II preliminary study	high-risk localized prostate cancer considered for neoadjuvant intra-arterial PSMA-targeted RLT	Not reported	Early-phase, feasibility-oriented design	[^177^Lu] Lu-PSMA-617	Neoadjuvant intra-arterial [^177^Lu] Lu-PSMA-617 before local definitive treatment	a single intravenous dose Dose escalation/de-escalation steps will be performed in accordance with the BOIN design	Adverse events related to intra-arterial, Days of delay of scheduled surgery due to adverse events related to intra-arterial

ADT, androgen deprivation therapy; BFS, biochemical failure-free survival; BOIN, Bayesian Optimal Interval; DLT, dose-limiting toxicity; EBRT, external beam radiotherapy; GBq, gigabecquerel; MTD, maximum tolerated dose; PET/CT, positron emission tomography/computed tomography; PSMA, prostate-specific membrane antigen; RLT, radioligand therapy; RP, radical prostatectomy; SBRT, stereotactic body radiotherapy; [177Lu], lutetium-177; [225Ac], actinium-225.

An important consideration emerging from these studies is that the combination of PSMA-targeted RLT with SBRT or EBRT may serve two distinct, although not mutually exclusive, therapeutic objectives. First, it may function as a locoregional intensification strategy aimed at improving primary tumour control. Second, it may provide systemic therapeutic benefit through the treatment of occult micro metastatic disease beyond the radiation field. These biologically distinct hypotheses should not be regarded as interchangeable and will require separate clinical validation.

## 4 Combination strategies with systemic treatment in PSMA-targeted radioligand therapy

Increasing attention has recently focused on combination strategies involving PSMA-targeted radioligand therapy and other systemic treatments, with the aim of enhancing treatment efficacy and overcoming mechanisms of therapeutic resistance.

These approaches are supported by several complementary biological mechanisms, including radiosensitisation, modulation of PSMA expression, improved targeting of tumour heterogeneity, and potential synergy through DNA damage response pathways. A broad range of therapeutic agents has been investigated in combination with PSMA-targeted RLT, including chemotherapy, androgen receptor pathway inhibitors (ARPIs), DNA damage repair inhibitors, immunotherapy, and other emerging targeted therapies.

However, the current evidence base remains heterogeneous, with most available data derived from early-phase clinical studies. Consequently, although these approaches are biologically attractive, their definitive clinical value remains to be established in larger prospective trials.

### 4.1 Modulation of androgen receptor signalling and PSMA expression

Combinations involving ARPIs represent one of the most biologically compelling strategies in PSMA-targeted RLT because androgen receptor signalling directly influences PSMA expression.

Androgens suppress expression of the *FOLH*1 gene, resulting in reduced PSMA messenger RNA transcription and lower PSMA protein expression (38). Conversely, androgen deprivation therapy (ADT) induces *FOLH*1 upregulation, leading to increased PSMA expression at the tumour cell surface (38). Both preclinical and clinical studies have demonstrated that treatment with ARPIs such as enzalutamide and abiraterone acetate is associated with increased PSMA expression, with expression levels subsequently returning towards baseline following treatment discontinuation. Importantly, this phenomenon has also been observed in tumour cells exhibiting resistance to antiandrogen therapy (38).

These observations provide a strong biological rationale for combining ARPIs with PSMA-targeted RLT, as increased target density may enhance radioligand uptake and potentially improve therapeutic efficacy.

The ENZA-p trial is an Australian multicentre, randomised, open-label phase II study evaluating an adaptive regimen of [^177^Lu]Lu-PSMA-617 combined with enzalutamide compared with enzalutamide alone as first-line treatment for patients with mCRPC exhibiting high-risk features for early progression (39). A total of 162 PSMA-positive patients, naïve to both docetaxel and ARPI therapy in the mCRPC setting, were enrolled. Patients received either enzalutamide (160 mg daily) alone or in combination with two to four cycles of 7.5 GBq [^177^Lu]Lu-PSMA-617 administered every six to eight weeks, with treatment adaptation guided by PSMA PET/CT findings at week 12The combination significantly improved PSA progression-free survival (13.0 vs. 7.8 months; HR 0.43; *p* < 0.0001), while also achieving higher PSA response rates (≥50% decline: 93% vs. 68%) and deeper PSA responses (≥90% decline: 78% vs. 37%) than enzalutamide monotherapy. Clinical progression-free survival and radiographic progression-free survival likewise favoured the combination arm.

Patient-reported outcomes also suggested greater improvement in pain among patients receiving combination therapy (61% vs. 27%). Treatment was generally well tolerated, with no unexpected toxicities observed and a safety profile broadly comparable to that reported with enzalutamide alone.

PSMA PET imaging substudies provided additional biological insight. The addition of [^177^Lu]Lu-PSMA-617 was associated with a significant reduction in PSMA-avid tumour burden, with a median decrease in PSMA-total tumour volume (PSMA-TTV) of 34% at three months. Residual PSMA-TTV at this time point emerged as a strong prognostic factor for overall survival, independent of treatment allocation and PSA response. Furthermore, the adverse prognostic effect associated with high baseline tumour burden in patients treated with enzalutamide alone appeared to be attenuated by the addition of [^177^Lu]Lu-PSMA-617 (40).

Although these findings are encouraging, interpretation should be tempered by the phase II design and the absence of mature overall survival data. Nevertheless, ENZA-p provides prospective clinical evidence supporting the concept that ARPI-mediated modulation of PSMA expression may enhance the activity of PSMA-targeted radioligand therapy. Among currently investigated combination approaches, ARPI-based strategies are supported by one of the strongest biological rationales and the most mature prospective clinical datasets available to date (40). The finals results of PSMA Addition trial will reinforce this strategy.

### 4.2 Chemotherapy-based combinations and radiosensitisation

The combination of PSMA-targeted radioligand therapy with chemotherapy represents one of the most extensively investigated approaches currently under evaluation. Several complementary biological mechanisms support this strategy. First, taxane-based agents such as docetaxel and cabazitaxel possess radiosensitising properties that may enhance radiation-induced DNA damage (41). Second, chemotherapy may target tumour cell populations with absent or low PSMA expression, thereby addressing a recognised limitation of PSMA-directed therapies. Finally, chemotherapy exerts systemic cytotoxic effects through mechanisms distinct from radiation, including suppression of tumour repopulation between treatment cycles and modulation of the tumour microenvironment, which may augment the overall antitumour effect of combined treatment.

Early clinical evidence supporting this approach was provided by a phase I study (NCT00916123) evaluating the combination of docetaxel with fractionated doses of [^177^Lu]Lu-J591, a monoclonal antibody directed against the extracellular domain of PSMA (42). Patients received docetaxel (75 mg/m^2^ every three weeks) in combination with escalating doses of [^177^Lu]Lu-J591 (1.48–2.96 GBq/m^2^). The regimen proved feasible and was associated with manageable haematological toxicity, without unexpected safety signals or dose-limiting toxicities. A PSA decline of at least 50% was observed in 73.3% of patients, while reductions in circulating tumour cell counts were also reported, indicating preliminary evidence of antitumour activity.

Building upon this early experience, the LuCAB trial (NCT05340374) was initiated as a phase I/II study evaluating the combination of cabazitaxel and [^177^Lu]Lu-PSMA-617 in patients with mCRPC (43). The study employs a dose-escalation design to determine the maximum tolerated dose of cabazitaxel when administered concurrently with up to six cycles of PSMA-targeted RLT. Secondary endpoints include biochemical response, radiographic progression-free survival, and overall survival.

Similarly, the LuCarbo trial (NCT06303713) is a phase I study investigating the combination of [^177^Lu]Lu-PSMA-617 with carboplatin in patients with mCRPC previously treated with docetaxel (44). The rationale for this combination derives from the potent DNA-damaging activity of platinum compounds and their potential to enhance radiation-induced cytotoxicity through complementary mechanisms of DNA damage.

Beyond conventional cytotoxic agents, alternative radiosensitising compounds have also been explored. The LuPIN trial evaluated the combination of [^177^Lu]Lu-PSMA-617 with idronoxil, a synthetic flavonoid with antiproliferative and antiangiogenic properties (45). In this heavily pretreated population, 61% of patients achieved a PSA decline of at least 50%, and median overall survival reached 19.7 months. Interpretation of these findings remains limited by the single-arm study design, the absence of a control group, and the potential influence of treatment-related toxicity associated with the route of administration.

Taken together, chemotherapy-based combinations are designed not only to enhance radiosensitisation of PSMA-expressing disease but also to target biologically aggressive or PSMA-low tumour subpopulations that may be less responsive to radioligand therapy alone.

While the biological rationale is strong, the clinical value of these approaches remains to be established. Their future integration into routine practice will depend on demonstrating meaningful efficacy gains without unacceptable cumulative toxicity, particularly with respect to bone marrow reserve and haematological adverse events.

### 4.3 DNA damage repair inhibition and PARP inhibitors

By emitting beta radiation, lutetium-177 induces DNA damage through the generation of ionisation events that result in both single-strand and double-strand DNA breaks. The exploitation of DNA damage response pathways has therefore emerged as an attractive strategy to enhance the efficacy of [^177^Lu]Lu-PSMA-617 (46).

Single-strand DNA breaks are primarily repaired through poly (ADP-ribose) polymerase (PARP)-dependent mechanisms. Inhibition of PARP promotes the accumulation of unrepaired DNA lesions, which may subsequently be converted into double-strand breaks during DNA replication. This provides a strong mechanistic rationale for combining PARP inhibitors (PARPi) with PSMA-targeted radioligand therapy (46).

The LuPARP trial (NCT03874884) is a phase I, open-label, dose-escalation study evaluating the safety and tolerability of olaparib in combination with [^177^Lu]Lu-PSMA-617 in patients with mCRPC previously treated with both ARPI therapy and docetaxel (47). The recommended phase II dose was established as olaparib 300 mg twice daily administered from day −4 to day +18 in combination with [^177^Lu]Lu-PSMA-617 at a dose of 7.4 GBq.

At this dose level, 55% of patients achieved a PSA90 response, while 88% of patients with measurable disease attained a partial radiological response. After a median follow-up of 17 months, median radiographic progression-free survival was 13.3 months and median overall survival was 33.5 months across the day −4 cohorts (DL7–9, *n* = 29) (47).

Although direct comparisons between studies should be avoided, these outcomes suggest that the combination warrants further evaluation in prospective clinical trials. Importantly, the phase I design and limited sample size preclude definitive conclusions regarding comparative efficacy (47).

In addition to evaluating clinical activity, translational analyses from LuPARP are expected to provide valuable insights into biomarkers of response and may help refine future patient-selection strategies beyond the current focus on BRCA alterations and homologous recombination repair deficiency.

Beyond PARP inhibition, other DNA damage response pathways are also being explored as therapeutic targets. DNA-dependent protein kinase (DNA-PK) plays a central role in the repair of radiation-induced double-strand DNA breaks through the non-homologous end-joining (NHEJ) pathway and is therefore considered an attractive radiosensitisation target in prostate cancer (48).

Inhibition of DNA-PK is expected to impair the repair of double-strand breaks induced by [^177^Lu]Lu-PSMA-617, thereby potentiating radiation-induced tumour cell death. On this basis, a phase Ib/II trial is currently evaluating the DNA-PK inhibitor AMO959 in combination with [^177^Lu]Lu-PSMA-617 and an androgen receptor pathway inhibitor in patients with PSMA-positive mCRPC (NCT07226986) (49).

Taken together, these approaches illustrate the growing interest in exploiting vulnerabilities within DNA damage response pathways to enhance the efficacy of PSMA-targeted radioligand therapy. While the biological rationale is compelling and early clinical data are encouraging, prospective studies will be required to determine whether these combinations translate into meaningful improvements in long-term clinical outcomes and to identify the patient populations most likely to benefit.

### 4.4 Immunotherapy and immune modulation

The combination of PSMA-targeted radioligand therapy with immunotherapy is based on the hypothesis that radiation may enhance tumour immunogenicity and promote antitumour immune responses (50). Radiation-induced cell death can increase tumour antigen presentation, stimulate dendritic cell activation, and modify the tumour microenvironment, potentially overcoming the immunologically “cold” phenotype that characterises most prostate cancers.

This biological rationale has prompted interest in combining PSMA-targeted RLT with immune checkpoint inhibitors (ICIs) in an attempt to enhance both the magnitude and durability of antitumour responses (50, 51). Despite encouraging preclinical observations, however, clinical evidence remains limited, and immune checkpoint inhibition as monotherapy has historically demonstrated modest activity in prostate cancer.

The phase Ib/II PRINCE study evaluated the combination of [^177^Lu]Lu-PSMA-617 and pembrolizumab in patients with mCRPC (52). The study enrolled 37 patients with a median age of 72 years, the majority of whom had previously received docetaxel (73%) and androgen receptor pathway inhibitors (100%). Patients received a median of five cycles of [^177^Lu]Lu-PSMA-617 and 12 doses of pembrolizumab, with a median follow-up of 16 months.

The PSA50 response rate was 76%, while a partial response according to RECIST criteria was observed in 70% of patients with measurable disease. Median radiographic progression-free survival was 11.2 months, and median overall survival was 17.8 months (52).

The combination was generally well tolerated. Most treatment-related adverse events were grade 1–2 and included xerostomia, fatigue, and dermatological toxicity. Grade 3 immune-related adverse events occurred in 27% of patients, although no dominant toxicity pattern was identified (52).

Interpretation of these findings is nevertheless limited by the absence of a control arm receiving PSMA-targeted RLT alone. Consequently, it remains uncertain whether the observed activity reflects a true synergistic interaction between radioligand therapy and pembrolizumab or whether outcomes were primarily driven by the effects of RLT itself.

A major limitation of the current evidence base is the lack of comparative data demonstrating that the addition of immune checkpoint inhibition improves outcomes beyond those expected with PSMA-targeted RLT alone. Whether radioligand therapy can meaningfully sensitise prostate tumours to immunotherapy through modulation of the tumour microenvironment, enhanced antigen presentation, or induction of systemic immune responses therefore remains an open question.

Further randomised studies will be required to clarify the role of immunotherapy in combination with PSMA-targeted RLT and to identify predictive biomarkers capable of selecting patients most likely to benefit from this approach.

## 5 Alternative PSMA-targeted Beta emitter

### 5.1 Terbium-161 (^161^Tb)

Terbium-161 (^161^Tb) has emerged as a promising radionuclide for next-generation PSMA-targeted radioligand therapy. It decays with a physical half-life of 6.95 days and emits *β*^−^ particles (average energy approximately 154 keV) together with *γ*-rays, notably at 48.9 keV (17.0%) and 74.6 keV (10.2%). These physical characteristics render ^161^Tb both chemically and physically comparable to lutetium-177 (^177^Lu). Its distinguishing feature, however, is the co-emission of internal conversion electrons and high-energy Auger electrons, which may increase cytotoxicity at the cellular and subcellular levels.

Preclinical studies have consistently demonstrated enhanced tumour cell killing and improved therapeutic efficacy of [^161^Tb]Tb-labelled radiopharmaceuticals compared with their [^177^Lu]-labelled counterparts, particularly in small tumour deposits and micrometastatic disease. This advantage has largely been attributed to the additional emission of conversion and Auger electrons, which increase local energy deposition while maintaining favourable tissue penetration characteristics (53).

Monte Carlo-based dosimetric simulations have further highlighted the microdosimetric properties of [^161^Tb]Tb. Compared with [^177^Lu]Lu, [^161^Tb]Tb delivers approximately two- to six-fold higher absorbed doses to cell membranes and two- to three-fold higher absorbed doses to cell nuclei. This enhanced energy deposition is principally driven by internal conversion and Auger electrons, which possess extremely short path lengths in biological tissues. Consequently, [^161^Tb]Tb may offer particular advantages for the treatment of minimal residual disease and occult micrometastases, including lesions that remain below the detection threshold of current molecular imaging techniques (54).

The Violet trial (NCT05521412) is a phase I/II study evaluating the safety, dosimetry, and preliminary efficacy of [^161^Tb]Tb-PSMA-I&T in patients with mCRPC. Thirty patients were enrolled between October 2022 and February 2024. Preliminary results indicate that [^161^Tb]Tb-PSMA-I&T is feasible and generally well tolerated. No dose-limiting toxicities were observed, and the recommended phase II dose was established at 7.4 GBq. With regard to treatment activity, 70% of patients achieved a PSA decline of at least 50%, while 40% achieved a PSA decline of at least 90%. Median radiographic progression-free survival was 11.1 months (55).

Additional clinical evidence has been generated through the prospective REALITY registry (NCT04833517), which reported the first clinical experience with [^161^Tb]Tb-PSMA-617 in heavily pretreated patients with mCRPC. Between September 2022 and February 2023, six patients received a single cycle of [^161^Tb]Tb-PSMA-617 with a mean administered activity of 6.4 GBq. Intra-individual dosimetric comparisons demonstrated a 2.4-fold higher tumour-absorbed dose with [^161^Tb]Tb-PSMA-617 than with [^177^Lu]Lu-PSMA-617, while maintaining favourable dosimetry for organs at risk, particularly the kidneys and salivary glands. Clinically, one patient achieved a partial response, three had stable disease, and two experienced disease progression. Treatment was generally well tolerated, with only mild haematological toxicity and xerostomia reported (56).

An expanded analysis of the REALITY registry subsequently included 18 patients treated with a total of 47 cycles of [^161^Tb]Tb-PSMA-617 and further supported the feasibility and safety of terbium-161-based PSMA-targeted radioligand therapy in heavily pretreated mCRPC. Signals of antitumour activity were also observed, although interpretation remains limited by the non-randomised nature of the registry and the small number of treated patients (57).

Taken together, the available preclinical, dosimetric, and early clinical data suggest that ^161^Tb may represent a potentially important development in beta-emitting PSMA-targeted radioligand therapy. Its capacity to deliver higher microscopic radiation doses while maintaining an apparently favourable toxicity profile has generated considerable interest, particularly in the context of small-volume disease, minimal residual disease, and micrometastatic tumour deposits. Nevertheless, whether these dosimetric advantages ultimately translate into clinically meaningful improvements in patient outcomes remains unknown. Prospective studies and direct comparative trials against established lutetium-based therapies will therefore be required before the precise clinical role of ^161^Tb can be defined.

## 6 Other non-PSMA target

### 6.1 Gastrin-releasing peptide receptor (GRPR)

The gastrin-releasing peptide receptor (GRPR), a G protein-coupled receptor belonging to the bombesin receptor family, has emerged as a potential alternative molecular target in prostate cancer. GRPR is expressed in several malignancies, including prostate cancer, and may represent a particularly relevant target in patients with absent, low, or heterogeneous PSMA expression.

A phase I study evaluated [^177^Lu]Lu-RM2, a GRPR-targeted radioligand, in patients with limited or absent PSMA expression. The primary objectives were to assess biodistribution, dosimetry, and preliminary safety characteristics of this therapeutic approach.

The highest physiological uptake of [^177^Lu]Lu-RM2 was observed in the pancreas, consistent with previous preclinical and clinical evidence demonstrating high GRPR expression in this organ. No significant uptake was detected in the salivary or lacrimal glands, suggesting a potentially favourable toxicity profile compared with PSMA-targeted radioligands, for which these tissues frequently represent dose-limiting organs (58).

At tumour sites, [^177^Lu]Lu-RM2 demonstrated rapid initial uptake, with the highest absorbed radiation doses observed in bone metastases, followed by lymph node and soft-tissue lesions. In contrast, hepatic metastases exhibited lower radioligand uptake. Substantial inter-patient variability in tumour-absorbed dose was also reported, likely reflecting heterogeneity of GRPR expression both between patients and across metastatic sites within individual patients (58).

Although clinical experience remains limited, these early observations suggest that GRPR-targeted radioligands may offer an alternative theranostic strategy for patients who are not optimal candidates for PSMA-targeted therapies. More importantly, they illustrate a broader principle increasingly recognised in prostate cancer theranostics: target heterogeneity may ultimately require the development of complementary molecular targets rather than reliance on a single biological pathway.

In this context, GRPR-based imaging and therapy should not necessarily be viewed as competitors to PSMA-directed approaches but rather as potentially complementary tools capable of expanding the spectrum of targetable prostate cancer phenotypes. Such strategies may become particularly relevant in advanced disease, where biological heterogeneity and variable target expression frequently emerge as mechanisms of treatment resistance.

Further clinical studies will be required to define the therapeutic efficacy of GRPR-targeted radioligands, identify the patient populations most likely to benefit, and clarify their place within the evolving landscape of precision radionuclide therapy for prostate cancer.

## 7 Discussion and further perspectives

Over the past decade, PSMA-targeted theranostics have transformed the management of advanced prostate cancer by establishing a paradigm in which molecular imaging and targeted radionuclide therapy are intrinsically linked. What initially emerged as a diagnostic biomarker evolved into a therapeutic platform capable of influencing treatment sequencing and refining patient selection, a shift validated by the pivotal phase III VISION trial. By demonstrating that [^177^Lu]Lu-PSMA-617 prolongs survival in PSMA-positive mCRPC, VISION confirmed that molecular imaging can identify therapeutic targets, determine eligibility, and enable systemic radiation delivery to biologically selected tumour sites—representing both a therapeutic and conceptual milestone that continues to reshape the interaction between nuclear medicine, medical oncology, radiation oncology, and urology.

As the field matures, the central question is no longer whether PSMA-targeted radioligand therapy is effective, but rather how it should be optimally integrated into clinical practice. Key uncertainties include patient selection, treatment sequencing, ligand selection, and the integration of RLT with other systemic therapies. In this context, SPLASH and ECLIPSE trials address an important unresolved issue: whether the clinical benefit observed with [^177^Lu]Lu-PSMA-617 reflects a class effect of lutetium-labelled PSMA ligands or whether clinically relevant differences exist between individual compounds. Variations in tumour uptake, pharmacokinetics, tumour residence time, organ dosimetry, and off-target exposure may ultimately influence efficacy and toxicity more than is currently recognised. The results of these studies are therefore likely to have implications extending beyond the individual radiopharmaceuticals being evaluated.

The expansion of PSMA-targeted RLT into earlier disease states represents a logical evolution of the field. However, earlier intervention should not automatically be assumed to confer greater clinical benefit. Patients treated in hormone-sensitive, oligometastatic, or neoadjuvant settings generally have longer life expectancy, more therapeutic options, and lower tolerance for treatment-related toxicity than patients with advanced mCRPC. Consequently, the evidentiary standards required for widespread adoption may be higher in these settings. Current studies consistently demonstrate encouraging signals of biological and clinical activity, improvements in PSA response rates and radiographic progression-free survival. Demonstration of meaningful improvements in overall survival, quality of life, treatment-free intervals, or long-term disease control will be required.

This consideration is particularly relevant in the contemporary therapeutic landscape, where patients may already receive multiple effective systemic and local treatments, including ADT, ARPIs, taxane-based chemotherapy, PARP inhibitors, stereotactic radiotherapy, and other targeted approaches. Earlier introduction of PSMA-targeted RLT may improve disease control, but it may also influence subsequent treatment sequencing, cumulative bone marrow toxicity, and therapeutic options available later in the disease course. The critical issue is therefore not whether RLT can be moved earlier, but whether doing so improves the overall trajectory of the disease.

Combination strategies constitute another major area of development. The biological rationale is compelling: ARPIs may increase PSMA expression, PARP inhibitors may impair repair of radiation-induced DNA damage, chemotherapy may target PSMA-low tumour populations, and immunotherapy may exploit radiation-induced immune modulation. Nevertheless, biological plausibility alone does not guarantee clinical benefit. The development of combination approaches will require careful integration of mechanistic understanding, biomarker-driven patient selection, and rigorous assessment of cumulative toxicity.

Among currently investigated strategies, combinations involving ARPIs appear the most clinically mature, supported by both mechanistic rationale and prospective clinical data. PARP inhibitor combinations are also particularly attractive because they directly exploit vulnerabilities in DNA damage response pathways. However, important questions remain regarding patient selection, optimal treatment schedules, and long-term haematological tolerance. Chemotherapy-based combinations may be particularly relevant in biologically heterogeneous disease, including tumours containing PSMA-low or PSMA-negative subclones, although their future role will depend on demonstrating that efficacy gains justify additional toxicity. By contrast, immunotherapy remains the least mature strategy. Although the underlying biological rationale is attractive, current evidence remains insufficient to establish that PSMA-targeted RLT consistently converts immunologically resistant prostate tumours into clinically meaningful responders to immune checkpoint inhibition.

The development of combination approaches will require careful integration of mechanistic understanding, biomarker-driven patient selection, and rigorous assessment of cumulative toxicity, particularly with respect to bone marrow reserve, as sequential or concurrent exposure to DNA-damaging agents may compound haematological adverse events.

### 7.1 Limitations and unresolved challenges

Despite the substantial progress achieved with PSMA-targeted beta-emitting radioligand therapy, several important limitations and unresolved challenges warrant dedicated consideration, as they may constrain the broader applicability and long-term success of this therapeutic paradigm.

### 7.2 Patient selection bias and trial generalizability

The clinical evidence supporting PSMA-targeted RLT derives predominantly from randomized controlled trials with stringent eligibility criteria that may not fully reflect real-world patient populations. The VISION trial, for instance, required PSMA positivity on central review and excluded patients with significant comorbidities, prior haematological malignancies, or impaired renal function. Similarly, the PSMAfore and SPLASH trials enriched for patients eligible for ARPI switching, implicitly selecting for slower disease progression and potentially more indolent phenotypes. These selection criteria may limit the generalizability of trial findings to broader clinical populations, including elderly patients with reduced performance status, those with extensive bone marrow involvement, or individuals with significant baseline cytopenias. Real-world evidence suggests that patients treated outside of clinical trial settings may experience different toxicity profiles and potentially attenuated efficacy, underscoring the importance of post-marketing surveillance and registry data to complement randomized evidence.

### 7.3 Heterogeneous PSMA expression, biological heterogeneity and treatment resistance

PSMA expression is neither uniform nor static across the disease continuum. Intrapatient heterogeneity, with some lesions demonstrating intense PSMA uptake while others show minimal or absent expression, represents a fundamental biological challenge. This heterogeneity may arise from clonal evolution under treatment pressure, with androgen receptor-independent subclones frequently exhibiting reduced PSMA expression. Discordant disease, characterised by FDG-positive/PSMA-negative lesions, has been consistently associated with treatment resistance and inferior clinical outcomes. The binary classification of patients as “PSMA-positive” or “PSMA-negative” based on visual assessment may oversimplify a continuous biological spectrum, potentially excluding patients who might derive partial benefit from RLT or including those with predominantly resistant subclones.

Resistance to PSMA-targeted RLT may emerge through multiple complementary mechanisms. Primary resistance may reflect pre-existing PSMA-low or PSMA-negative tumour subpopulations that are inherently refractory to radioligand delivery. Acquired resistance may develop through the outgrowth of resistant clones following treatment-induced selective pressure, analogous to mechanisms observed with targeted systemic therapies. Additional resistance mechanisms include inadequate radiation dose delivery in large tumour deposits or marrow-predominant disease, where beta particle range may be insufficient for complete cytoreduction; intrinsic radioresistance mediated by enhanced DNA repair capacity; and pharmacokinetic factors such as rapid radioligand clearance or competitive binding. Failure following radioligand therapy may therefore reflect not only insufficient radiation delivery but also the persistence or selection of resistant tumour clones lacking adequate target expression.

These observations underscore the evolving role of molecular imaging as a biomarker of tumour biology. Quantitative imaging parameters, including whole-body tumour burden, SUVmean, lesion heterogeneity, FDG discordance, and early volumetric response, may ultimately provide more clinically relevant information than binary classifications of PSMA positivity. Future patient-selection strategies will likely rely on integrated biological and imaging phenotypes rather than simple threshold-based approaches.

### 7.4 Economic limitations and access disparities

The widespread implementation of PSMA-targeted theranostics is constrained by substantial economic and logistical barriers. The production of [^177^Lu]Lu-PSMA-617 requires dedicated radiopharmacy infrastructure, cyclotron or generator-based radionuclide supply, and specialised handling facilities that may be unavailable in resource-limited settings. The cost of radioligand therapy, including the radiopharmaceutical itself, associated imaging, and hospitalisation for administration, represents a significant healthcare expenditure that may limit access in lower-income regions and healthcare systems with restricted reimbursement frameworks. Disparities in access are further compounded by uneven geographic distribution of nuclear medicine facilities, with rural and underserved populations facing prolonged travel times and reduced treatment availability. As the field expands toward earlier disease stages requiring multiple treatment cycles over extended periods, the cumulative economic burden will increase, necessitating health technology assessment and value-based reimbursement frameworks to ensure equitable access.

### 7.5 Toxicity profiles across clinical trials

The safety profile of PSMA-targeted beta-emitting radioligand therapy has been characterised across multiple phase III clinical trials, revealing a generally predictable and manageable spectrum of adverse events, with important variations across treatment settings and compounds.

In the VISION trial, [^177^Lu]Lu-PSMA-617 was associated with a higher incidence of grade 3–5 adverse events compared with standard of care alone (52.7% vs. 38.0%), with treatment-related grade 3–4 adverse events occurring in 28.4% of patients receiving radioligand therapy vs. 3.9% in the control arm. The most common adverse events in the [^177^Lu]Lu-PSMA-617 arm included fatigue (43%), dry mouth (39%), nausea (35%), anaemia (32%), and thrombocytopenia (17%). Five treatment-related deaths occurred in the radioligand therapy arm (0.9%), attributed to pancytopenia, bone marrow failure, and haemorrhagic complications. Importantly, when compared with taxane-based chemotherapy in the TheraP trial, [^177^Lu]Lu-PSMA-617 demonstrated a more favourable toxicity profile, with grade 3–4 adverse events occurring in 33% vs. 53% of patients treated with cabazitaxel.

In the PSMAfore trial, evaluating [^177^Lu]Lu-PSMA-617 in the pre-taxane setting, grade 3–5 adverse events occurred in 36% of patients receiving radioligand therapy, with dry mouth reported in 59.5% of patients—a higher rate than observed in VISION (38.8%), potentially attributable to more robust adverse event reporting and a median of six treatment cycles vs. five in VISION. Haematologic adverse events were more frequent with [^177^Lu]Lu-PSMA-617 than with ARPI change (39.2% vs. 23.2%), with higher rates observed in patients with extensive bone metastases, suggesting bone marrow irradiation as a contributing mechanism. Renal toxicity was low and comparable between treatment arms (<10%), with rapid resolution (median 0.71 months).

The SPLASH trial, evaluating [^177^Lu]Lu-PSMA-I&T (PNT2002) at a lower per-cycle dose (6.8 GBq every 8 weeks for up to four cycles) compared with PSMAfore (7.4 GBq every 6 weeks for up to six cycles), reported grade ≥ 3 treatment-emergent adverse events in 30.1% of patients. Notable differences between compounds included a lower incidence of dry mouth with [^177^Lu]Lu-PSMA-I&T (37%) compared with [^177^Lu]Lu-PSMA-617 (58% in PSMAfore), whereas fatigue was more frequently reported in SPLASH (54%) than in PSMAfore (23%). Dosimetric analyses suggest higher salivary gland exposure with PSMA-617 and higher renal exposure with PSMA-I&T, providing a mechanistic basis for these divergent toxicity profiles.

In the hormone-sensitive setting, preliminary data from the PSMAddition trial indicate that the addition of [^177^Lu]Lu-PSMA-617 to standard ADT plus ARPI was generally well tolerated, with no unexpected safety signals and a toxicity profile consistent with that observed in the mCRPC setting. Similarly, in the UpFrontPSMA trial, sequential [^177^Lu]Lu-PSMA-617 followed by docetaxel did not result in additive haematological toxicity beyond that expected with either agent alone.

Across trials, the principal safety considerations include myelosuppression (particularly in patients with extensive bone disease), xerostomia (generally mild-to-moderate and reversible), and rare but serious events including bone marrow failure and haemorrhagic complications. The observation that grade 3 or higher haematologic toxicity was higher in VISION (post-taxane setting) than in PSMAfore (pre-taxane setting) supports the hypothesis that earlier administration of PSMA-targeted RLT, before cumulative bone marrow compromise from prior therapies, may improve the therapeutic index.

The development of terbium-161 further illustrates the continuing evolution of radionuclide therapy. The additional conversion and Auger electron emissions associated with ^161^Tb provide compelling dosimetric advantages, particularly for micrometastatic disease and small-volume tumour deposits. These characteristics are especially relevant as radioligand therapy moves towards earlier disease stages. Nevertheless, the relationship between favourable microdosimetry and clinical benefit remains to be demonstrated. The future role of ^161^Tb will therefore depend on prospective evidence showing that these physical advantages translate into meaningful improvements in patient outcomes. Alpha emitters such as Ac225, Lead 212 or At211 that are not in the scope of this review, also represent the next step to improve PSMA RLT efficacy even if some concerns about toxicity are under investigation.

Similarly, the investigation of non-PSMA targets such as GRPR reflects an increasingly important recognition that prostate cancer is biologically heterogeneous and may not be adequately addressed through a single molecular target. Alternative or complementary targets may become particularly relevant in patients with low PSMA expression, treatment-emergent target loss, or mixed biological phenotypes. In this respect, future theranostic strategies may increasingly incorporate complementary imaging biomarkers, sequential targeting approaches, or multi-target radionuclide therapies.

Taken together, PSMA-targeted beta-emitting radioligand therapy has evolved beyond its initial role as a late-line treatment for mCRPC. It is increasingly becoming an adaptable therapeutic platform situated at the intersection of molecular imaging, radiation biology, systemic therapy, and tumour evolution. Future progress will depend less on demonstrating efficacy—which has already been established in selected populations—and more on refining how, when, and in whom these therapies should be deployed.

The next stage of development will therefore require a wider precision-medicine approach. Careful patient selection, rational treatment sequencing, biologically informed combinations, and a deeper understanding of resistance mechanisms will be essential to preserve and maximise the therapeutic index of PSMA-targeted RLT. If these challenges can be addressed successfully, beta-emitting theranostics may become one of the central components of prostate cancer management across multiple stages of disease. Figure 1

**Figure 1:**
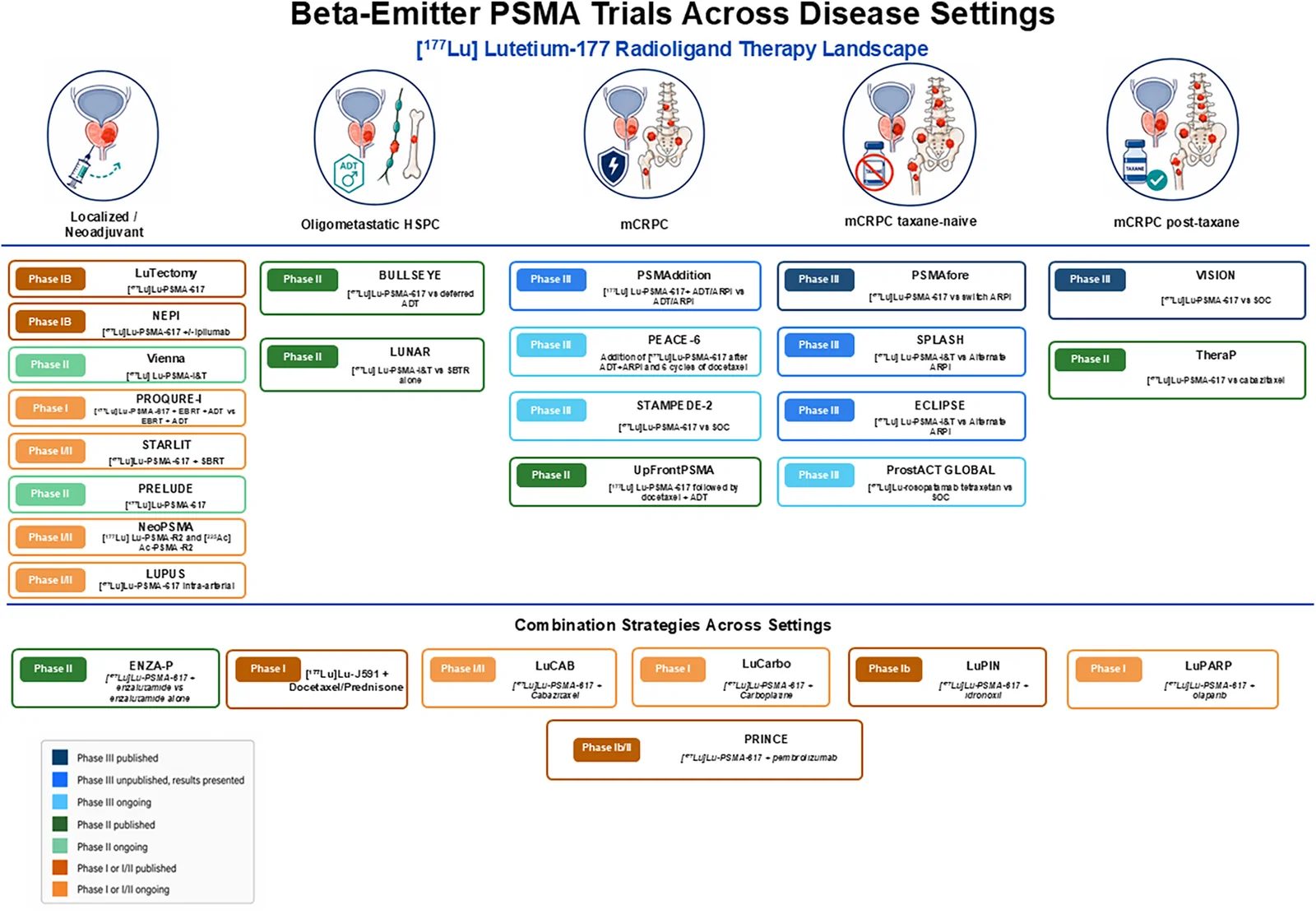
Landscape of beta-emitter PSMA radioligand therapy trials across the prostate cancer disease continuum.

## 8 Integrated perspective

The evolution of PSMA-targeted radioligand therapy represents more than the introduction of a new treatment modality. It reflects a broader shift in the management of prostate cancer, from treatment strategies primarily defined by clinical stage and disease burden towards approaches increasingly guided by tumour biology and molecular imaging.

Historically, therapeutic decisions in prostate cancer were largely determined by anatomical extent of disease, prior treatments, and clinical classification. The theranostic paradigm introduces a different framework in which treatment selection is informed by molecular target expression, tumour phenotype, and functional imaging characteristics. In this model, imaging is no longer confined to disease detection and staging but becomes an integral component of therapeutic decision-making.

The clinical success of [^177^Lu]Lu-PSMA-617 in mCRPC demonstrated that systemic radiation can be delivered with sufficient tumour selectivity to improve clinically meaningful outcomes while maintaining an acceptable safety profile. However, the significance of this achievement extends beyond a single radiopharmaceutical. The field is progressively evolving from a drug-centred approach towards a broader therapeutic platform in which different ligands, radionuclides, molecular targets, and combination strategies may coexist within a common theranostic framework.

In this context, future progress is likely to depend less on identifying a universally superior radiopharmaceutical than on understanding how specific biological contexts require different therapeutic approaches. Consequently, the clinically relevant question may no longer be which radioligand performs best overall, but rather which radioligand is most appropriate for a particular patient, disease stage, and biological phenotype.

Such an evolution implies a gradual transition from standardised treatment algorithms towards more individualised therapeutic strategies. Patients with low-volume hormone-sensitive disease, oligometastatic recurrence, and heavily pretreated mCRPC may all demonstrate PSMA expression, yet the therapeutic objectives differ substantially across these settings. In one context, treatment may aim to eradicate microscopic residual disease; in another, to delay progression; and in advanced disease, to control resistant tumour populations while preserving quality of life. The same radionuclide may therefore fulfil different therapeutic roles depending on the biological and clinical context in which it is applied.

This perspective also highlights a central challenge within contemporary theranostics. The success of PSMA-targeted approaches has been driven by patient selection based on target expression, yet the biological heterogeneity of prostate cancer increasingly exposes the limitations of a single-target strategy. PSMA expression is dynamic rather than fixed. It varies between lesions, evolves under treatment pressure, and may diminish as resistant or dedifferentiated tumour clones emerge. Consequently, future advances are unlikely to depend exclusively on increasingly sophisticated PSMA-directed approaches but may require the integration of additional molecular targets capable of capturing a broader spectrum of tumour biology.

The emergence of GRPR-targeted radioligands and other non-PSMA approaches illustrates this evolution. Rather than being viewed as competitors to PSMA-directed therapies, these agents may ultimately complement existing strategies by addressing tumour phenotypes characterised by low PSMA expression, target heterogeneity, or treatment-emergent biological divergence. Future theranostic pathways may therefore incorporate complementary imaging biomarkers, sequential molecular targeting, or multi-target radionuclide approaches tailored to individual tumour characteristics.

Advances in molecular imaging are likely to drive a similar evolution in patient selection. Current treatment paradigms remain largely binary, classifying patients according to the presence or absence of PSMA expression. Increasing evidence suggests, however, that quantitative and biological imaging characteristics—including tumour burden, intrapatient heterogeneity, SUV-derived metrics, FDG discordance, and dynamic treatment-induced changes—may provide more clinically relevant information than simple target positivity alone. Future decision-making will likely rely on integrated imaging phenotypes rather than isolated imaging findings.

A parallel development concerns the radionuclides themselves. Lutetium-177 has become the current reference standard because it achieves a favourable balance between efficacy and safety. Nevertheless, the development of terbium-161 illustrates how radionuclide therapy continues to evolve towards agents with distinct microdosimetric characteristics and potentially greater activity against microscopic disease. Rather than replacing lutetium-177, future radionuclides may occupy complementary roles according to tumour burden, lesion size, biological aggressiveness, and therapeutic objectives. Such an approach would represent a transition from radionuclide substitution to radionuclide selection based on biological context.

Perhaps the most important implication of the theranostic paradigm is its potential to influence treatment sequencing. Traditionally, prostate cancer therapies have been organised according to successive lines of treatment. Theranostics introduces the possibility of selecting treatments according to biological vulnerability identified through molecular imaging. In this framework, imaging may become a dynamic tool capable of characterising dominant tumour phenotypes over time and guiding treatment selection accordingly.

Ultimately, the future of PSMA-targeted beta-emitting radioligand therapy is unlikely to depend on a single breakthrough ligand, radionuclide, or combination regimen. Long-term progress will instead depend on the ability to match the appropriate target, radionuclide, ligand, and timing of intervention to the biological characteristics of each individual patient. The most durable advances are therefore likely to arise from greater biological precision rather than greater therapeutic intensity.

In this respect, the impact of beta-emitting theranostics may extend beyond prostate cancer itself. It provides a practical example of an oncology model in which diagnosis and therapy are integrated within a single biologically informed strategy, with molecular imaging serving not only to identify disease but also to guide therapeutic intervention throughout the course of treatment.

## 9 Conclusion

PSMA-targeted radioligand therapy has become one of the most important therapeutic advances in prostate cancer over the past decade. The demonstration that [^177^Lu]Lu-PSMA-617 improves both radiographic progression-free survival and overall survival in patients with metastatic castration-resistant prostate cancer established targeted beta irradiation as a clinically effective treatment and validated the theranostic paradigm in routine practice.

The progress achieved in this field extends beyond the development of a single radiopharmaceutical. Rather, it reflects the emergence of an integrated therapeutic model in which molecular imaging, tumour biology, patient selection, and systemic treatment are closely interconnected. As a result, PSMA-targeted radioligand therapy has evolved from a novel treatment approach into a broader clinical platform capable of guiding both therapeutic decision-making and treatment delivery.

The evidence reviewed in this article highlights several developments that are reshaping prostate cancer theranostics. PSMA PET has become central to patient selection, prognostic assessment, and treatment monitoring. Radioligand therapy is being evaluated across progressively earlier disease states, including hormone-sensitive, oligometastatic, and neoadjuvant settings. Combination strategies involving androgen receptor pathway inhibitors, DNA damage repair inhibitors, chemotherapy, immunotherapy, and local ablative treatments are being explored to address tumour heterogeneity and mechanisms of treatment resistance. In parallel, the development of alternative radionuclides such as terbium-161 and emerging molecular targets including GRPR reflects the continuing expansion of the theranostic landscape.

Despite these advances, several important challenges remain, including patient selection bias inherent to clinical trial populations, biological heterogeneity and treatment resistance, the management of PSMA-negative disease, the imperfect specificity of molecular imaging, the economic and logistical barriers to widespread implementation, and the long-term consequences of treatment intensification. The optimal positioning of radioligand therapy within increasingly complex treatment pathways has yet to be defined. As radioligand therapy moves towards earlier stages of disease, demonstration of meaningful benefits in overall survival, quality of life, and durable disease control will become increasingly important.

Furthermore, alpha-emitting radioligands such as [^225^Ac]Ac-PSMA-617, which deliver high linear energy transfer radiation with a short tissue range, offer a promising avenue for patients who have progressed on beta-emitting therapy, potentially overcoming resistance mediated by reduced PSMA expression or inadequate dose deposition in resistant clones. Several alpha-emitting constructs are currently under clinical investigation, reflecting substantial industry interest in targeted alpha therapy, although the limited global supply of actinium-225 remains a significant constraint.

Future progress will depend less on expanding indications and more on refining patient selection. The ability to integrate molecular imaging, tumour phenotype, radionuclide characteristics, and treatment sequencing into biomarker-driven strategies is likely to determine the long-term success of the field.

PSMA-targeted theranostics is no longer confined to a single radioligand or a single stage of disease. It is evolving into a precision-oncology framework in which imaging and therapy are intrinsically linked. The next phase of development will depend on identifying the right target, the right radiopharmaceutical, and the right therapeutic timing for each patient. In this setting, the greatest advances are likely to arise not from greater treatment intensity, but from greater biological precision.

## References

[1] Bray F Laversanne M Sung H Ferlay J Siegel RL Soerjomataram I. Global cancer statistics 2022: GLOBOCAN estimates of incidence and mortality worldwide for 36 cancers in 185 countries. CA Cancer J Clin. (2024) 74(3):229–63. 10.3322/caac.21834 38572751

[2] Sung H Filho AMR Laversanne M Ferlay J Siegel RL Soerjomataram I. Global cancer statistics 2024: GLOBOCAN estimates of incidence and mortality worldwide for 34 cancers in 186 countries. CA Cancer J Clin. (2026) 74(3):229–63. 10.3322/caac.21834 PMC13343830 42417444

[3] Kratochwil C Fendler WP Eiber M Hofman MS Emmett L Calais J. Joint EANM/SNMMI procedure guideline for the use of [^177^Lu]-labeled PSMA-targeted radioligand therapy ([^177^Lu]-PSMA-RLT). Eur J Nucl Med Mol Imaging. (2023) 50(9):2830–45. 10.1007/s00259-023-06255-8 37246997 PMC10317889

[4] Sartor O De Bono J Chi KN Fizazi K Herrmann K Rahbar K. [^177^lu]lu-PSMA-617 for metastatic castration-resistant prostate cancer. N Engl J Med. (2021) 385(12):1091–103. 10.1056/NEJMoa2107322 34161051 PMC8446332

[5] Wang B Liu C Wei Y Meng J Zhang Y Gan H. A prospective trial of ^68^Ga-PSMA and ^18^F-FDG PET/CT in nonmetastatic prostate cancer patients with an early PSA progression during castration. Clin Cancer Res. (2020) 26(17):4551–8. 10.1158/1078-0432.CCR-20-0580 32527944

[6] Hofman MS Violet J Hicks RJ Ferdinandus J Thang SP Akhurst T. [^177^lu]lu-PSMA-617 radionuclide treatment in patients with metastatic castration-resistant prostate cancer (LuPSMA trial): a single-centre, single-arm, phase 2 study. Lancet Oncol. (2018) 19(6):825–33. 10.1016/S1470-2045(18)30198-0 29752180

[7] Buteau J Martin AJ Emmett L Violet J Sandhu S Iravani A. PSMA and FDG PET as predictive and prognostic biomarkers in patients given [^177^Lu]lu-PSMA-617 versus cabazitaxel for metastatic castration-resistant prostate cancer (TheraP): a biomarker analysis from a randomized, open-label, phase 2 trial. Lancet Oncol. (2022) 23(11):1389–97. 10.1016/S1470-2045(22)00519-6 36261050

[8] Eiber M Herrmann K Calais J Hadaschik B Giesel FL Hartenbach M. Prostate cancer molecular imaging standardized evaluation (PROMISE): proposed miTNM classification for the interpretation of PSMA-ligand PET/CT. J Nucl Med. (2018) 59(3):469–78. 10.2967/jnumed.117.198119 29123012

[9] Seifert R Emmett L Rowe SP Herrmann K Hadaschik B Calais J. Second version of the prostate cancer molecular imaging standardized evaluation framework including response evaluation for clinical trials (PROMISE V2). Eur Urol. (2023) 83(5):405–12. 10.1016/j.eururo.2023.02.002 36935345

[10] Werner RA Hartrampf PE Fendler WP Serfling SE Derlin T Higuchi T. Prostate-specific membrane antigen reporting and data system version 2.0. Eur Urol. (2023) 84(5):491–502. 10.1016/j.eururo.2023.06.008 37414701 PMC11981304

[11] Rafiei Alavi N Rahmani M Gupta A Yaghmai V. PSMA-RADS to improve evaluation and management of prostate cancer. Abdom Radiol. (2025) 50(7):3272–83. 10.1007/s00261-024-04738-2 39702798

[12] Eisenhauer EA Therasse P Bogaerts J Schwartz LH Sargent D Ford R. New response evaluation criteria in solid tumours: revised RECIST guideline (version 1.1). Eur J Cancer. (2009) 45(2):228–47. 10.1016/j.ejca.2008.10.026 19097774

[13] Scher HI Morris MJ Stadler WM Higano C Basch E Fizazi K. Trial design and objectives for castration-resistant prostate cancer: updated recommendations from the prostate cancer clinical trials working group 3. J Clin Oncol. (2016) 34(12):1402–18. 10.1200/JCO.2015.64.2702 26903579 PMC4872347

[14] Gafita A Rauscher I Weber M Hadaschik B Wang H Armstrong WR. Novel framework for treatment response evaluation using PSMA PET/CT in patients with metastatic castration-resistant prostate cancer (RECIP 1.0): an international multicenter study. J Nucl Med. (2022) 63(11):1651–8. 10.2967/jnumed.121.263072 35422442 PMC9635677

[15] Gafita A Djaileb L Rauscher I Fendler WP Hadaschik B Rowe SP. Response evaluation criteria in PSMA PET/CT (RECIP 1.0) in metastatic castration-resistant prostate cancer. Radiology. (2023) 308(1):e222148. 10.1148/radiol.222148 37432081 PMC10374938

[16] Armstrong AJ Morris MJ Abida W Aggarwal RR Antonarakis ES Attard G. Trial design and objectives for patients with prostate cancer: recommendations from the prostate cancer working group 4. J Clin Oncol. (2026) 44(11):1249–65. 10.1200/JCO-25-02834 41744290 PMC13132095

[17] Herrmann K Walz J MacLennan S Briganti A Cornford P Czernin J. SPARC: the standardised prostate-specific membrane antigen positron emission tomography/computed tomography analysis and reporting consensus: a delphi analysis. Eur Urol. (2026) 89(3):260–74. 10.1016/j.eururo.2025.08.005 40945999

[18] Hofman MS Emmett L Sandhu S Iravani A Buteau JP Joshua AM. Overall survival with [^177^Lu]lu-PSMA-617 versus cabazitaxel in metastatic castration-resistant prostate cancer (TheraP): secondary outcomes of a randomized, open-label, phase 2 trial. Lancet Oncol. (2024) 25(1):99–107. 10.1016/S1470-2045(23)00529-6 38043558

[19] Morris MJ Castellano D Herrmann K De Bono JS Shore ND Chi KN. [^177^lu]lu-PSMA-617 versus a change of androgen receptor pathway inhibitor therapy for taxane-naïve patients with progressive metastatic castration-resistant prostate cancer (PSMAfore): a phase 3, randomized, controlled trial. Lancet. (2024) 404(10459):1227–39. 10.1016/S0140-6736(24)01653-2 39293462 PMC12121614

[20] Hansen AR Probst S Beauregard J-M Viglianti BL Michalski JM Tagawa ST. Initial clinical experience with [^177^Lu]lu-PNT2002 radioligand therapy in metastatic castration-resistant prostate cancer: dosimetry, safety, and efficacy from the lead-in cohort of the SPLASH trial. Front Oncol. (2025) 14:1483953. 10.3389/fonc.2024.1483953 39839782 PMC11745944

[21] ClinicalTrials.gov. ECLIPSE: a randomized, open-label, phase 3 trial comparing [^177^Lu]Lu-PSMA-I&T to hormone therapy in patients with PSMA-positive metastatic castration-resistant prostate cancer (mCRPC). Identifier: NCT05204927. Available online at: https://clinicaltrials.gov/study/NCT05204927 (Accessed September 8, 2026).

[22] Sartor AO Bögemann M Borre M Carles J Choyke PL Heidenreich A. A phase 2/3 study of [^177^Lu]lu-TLX591 plus standard of care versus standard of care alone in patients with metastatic castration-resistant prostate cancer (ProstACT GLOBAL). Int J Radiat Oncol Biol Phys. (2024) 120(2):e83. 10.1016/j.ijrobp.2024.07.008

[23] Sartor AO Tagawa ST Saad F De Bono JS Feng FY Fizazi K. PSMAddition: a phase 3 trial to compare treatment with [^177^Lu]lu-PSMA-617 plus standard of care versus standard of care alone in patients with metastatic hormone-sensitive prostate cancer (mHSPC). J Clin Oncol. (2022) 40(6_suppl):TPS210. 10.1200/JCO.2022.40.6_suppl.TPS210

[24] Tagawa ST Sartor O Piulats JM Fizazi K Tombal B Chi KN. Phase III trial of [177Lu]Lu-PSMA-617 combined with ADT plus ARPI in patients with PSMA-positive metastatic hormone-sensitive prostate cancer (PSMAddition). Presented at: ESMO Congress 2025; Presidential Symposium; Abstract LBA6; October 19, 2025; Berlin, Germany.

[25] Fizazi K Gravis G Latorzeff I Ezziddin S Haberkorn U Rahbar K. PEACE-6 Poor Responders: a randomized phase III trial evaluating the efficacy and safety of [^177^Lu]Lu-PSMA-617 plus standard of care versus standard of care alone in patients with *de novo* metastatic hormone-sensitive prostate cancer with serum PSA ≥0.2 ng/mL after 6–8 months of systemic therapy. Protocol UC-GTG-2301 (GETUG-AFU 42). UNICANCER; 2024. Available online at: ClinicalTrials.gov, NCT06496581.

[26] ClinicalTrials.gov. STAMPEDE-2: A randomized controlled platform trial testing novel treatments added to androgen deprivation therapy in metastatic hormone-sensitive prostate cancer (STAMPEDE-2). Identifier: NCT06320067. Available online at: https://clinicaltrials.gov/study/NCT06320067 (Accessed September 8, 2026).

[27] Azad A Bressel M Tan H Violet J Wong LM Emmett L. Sequential [^177^Lu]lu-PSMA-617 and docetaxel versus docetaxel in patients with metastatic hormone-sensitive prostate cancer (UpFrontPSMA): a multicentre, open-label, randomized, phase 2 study. Lancet Oncol. (2024) 25(10):1267–76. 10.1016/S1470-2045(24)00384-7 39293461

[28] Privé BM Noordzij W Muselaers CHJ De Jong IJ Van Oort IM Janssen MJR. [^177^lu]lu-PSMA-617 in oligometastatic hormone-sensitive prostate cancer (BULLSEYE): an open-label, randomised, phase 2 study. Lancet Oncol. (2026) 27(4):461–9. 10.1016/S1470-2045(25)00762-4 41926960

[29] Ma TM Czernin J Felix C Alano R Wilhalme H Valle L. LUNAR: a randomized phase 2 study of [^177^Lu]lu-PSMA neoadjuvant to ablative radiotherapy for oligorecurrent prostate cancer (clinical trial protocol). BJU Int. (2023) 132(1):65–74. 10.1111/bju.15988 36797449

[30] Kishan AU Valle LF Wilhalme H Felix C Nabong R Juarez-Casillas JE. [^177^lu]lu-prostate-specific membrane antigen neoadjuvant to stereotactic ablative radiotherapy for oligorecurrent prostate cancer (LUNAR): an open-label, randomized, controlled, phase II study. J Clin Oncol. (2025) 43(36):3812–21. 10.1200/JCO-25-01553 41223345 PMC12711279

[31] Eapen RS Buteau JP Jackson P Mitchell C Oon SF Alghazo O. Administering [^177^Lu]lu-PSMA-617 prior to radical prostatectomy in men with high-risk localized prostate cancer (LuTectomy): a single-centre, single-arm, phase 1/2 study. Eur Urol. (2024) 85(3):217–26. 10.1016/j.eururo.2023.08.026 37891072

[32] ClinicalTrials.gov. Neoadjuvant Lu-PSMA radioligand therapy and ipilimumab in men with very high-risk prostate cancer (NEPI). Identifier: NCT06388369. Available online at: https://clinicaltrials.gov/study/NCT06388369 (Accessed September 8, 2026).

[33] ClinicalTrials.gov. Neoadjuvant PSMA-radioligand therapy in oligometastatic prostate cancer. Phase II trial. Identifier: NCT06259123. Available online at: https://clinicaltrials.gov/study/NCT06259123 (Accessed September 8, 2026).

[34] ClinicalTrials.gov. EBRT plus Lu-PSMA for N1M0 prostate cancer (PROQURE-1). Phase I trial. Identifier: NCT05162573. Available online at: https://clinicaltrials.gov/study/NCT05162573 (Accessed September 8, 2026).

[35] ClinicalTrials.gov. STereotActic Body Radiotherapy and ^177^Lu-PSMA-617 for Locally Advanced Prostate Cancer (STARLiT Trial). Identifier: NCT06574880. Available online at: https://clinicaltrials.gov/study/NCT06574880 (Accessed September 8, 2026).

[36] ClinicalTrials.gov. Neoadjuvant [^177^Lu]Lu-PSMA-617 in Patients with High-Risk Localized Prostate Cancer Undergoing Radical Prostatectomy (PRELUDE Trial). Identifier: NCT06798558. Available online at: https://clinicaltrials.gov/study/NCT06798558 (Accessed September 8, 2026).

[37] LUPUS Study Investigators. Safety and feasibility of neoadjuvant intra-arterial [^177^Lu]Lu-vipivotide tetraxetan in high-risk localized prostate cancer: preliminary results of the phase I/II LUPUS trial. Presented at: European Association of Urology Congress (EAU); 2025 March 21–24; Madrid, Spain.

[38] Vaz S Hadaschik B Gabriel M Herrmann K Eiber M Costa D. Influence of androgen deprivation therapy on PSMA expression and PSMA-ligand PET imaging of prostate cancer patients. Eur J Nucl Med Mol Imaging. (2020) 47(1):9–15. 10.1007/s00259-019-04529-8 31654093

[39] Emmett L Subramaniam S Crumbaker M Nguyen A Joshua AM Weickhardt A. [^177^lu]lu-PSMA-617 plus enzalutamide in patients with metastatic castration-resistant prostate cancer (ENZA-p): an open-label, multicentre, randomized, phase 2 trial. Lancet Oncol. (2024) 25(5):563–71. 10.1016/S1470-2045(24)00135-9 38621400

[40] Subramaniam S Emmett L Crumbaker M. Prognostic value of 3-month PSMA-PET total tumor volume in ENZA-p: a randomized phase 2 trial of [^177^Lu]lu-PSMA-617 plus enzalutamide versus enzalutamide in metastatic castration-resistant prostate cancer. J Nucl Med. (2026) 67(1):112–20. 10.2967/jnumed.125.269252

[41] Golden EB Formenti SC Schiff PB. Taxanes as radiosensitizers. Anticancer Drugs. (2014) 25(5):502–11. 10.1097/CAD.0000000000000055 24335716

[42] Batra JS Niaz MJ Whang YE Sheikh A Thomas C Christos P. Phase 1 trial of docetaxel plus [^177^Lu]lu-J591 (lutetium-177-labeled anti-prostate-specific membrane antigen monoclonal antibody) for metastatic castration-resistant prostate cancer. Urol Oncol. (2020) 38(11):848.e9–848.e16. 10.1016/j.urolonc.2020.05.028 32600929

[43] Kostos L Buteau JP Kong G Tran B Haskali MB Fahey M. Clinical trial protocol for LuCAB: a phase 1–2 trial evaluating cabazitaxel in combination with [177Lu]lu-PSMA-617 in patients with metastatic castration-resistant prostate cancer. J Nucl Med. (2025) 66(4):572–8. 10.2967/jnumed.124.269252 39978808

[44] ClinicalTrials.gov. LuCarbo: A phase 1a/1b study of ^177^Lu-PSMA-617 plus carboplatin in metastatic castrate-resistant prostate cancer. Identifier: NCT06303713. Available online at: https://clinicaltrials.gov/study/NCT06303713 (Accessed September 8, 2026).

[45] Pathmanandavel S Crumbaker M Yam AO Nguyen A Rofe C Hovey E. [^177^lu]lu-PSMA-617 and idronoxil in men with end-stage metastatic castration-resistant prostate cancer (LuPIN): patient outcomes and predictors of treatment response in a phase 1–2 trial. J Nucl Med. (2022) 63(4):560–6. 10.2967/jnumed.121.262552 34326127 PMC8973290

[46] Monfared Y K Heidari P Klempner SJ Mahmood U Parikh AR Hong TS. DNA damage by radiopharmaceuticals and mechanisms of cellular repair. Pharmaceutics. (2023) 15(12):2761. 10.3390/pharmaceutics15122761 38140100 PMC10748326

[47] Sandhu SK Joshua AM Emmett L. LuPARP: phase I trial of [^177^Lu]lu-PSMA-617 and olaparib in patients with metastatic castration resistant prostate cancer (mCRPC). Ann Oncol. (2025) 36(suppl 5):2391P.

[48] Wang XH Wang ZQ Mu ZY Zhu LP Zhong CF Guo S. The efficacy and safety of immune checkpoint inhibitors in metastatic castration-resistant prostate cancer: a systematic review and meta-analysis. Medicine (Baltimore. (2022) 101(31):e29715. 10.1097/MD.0000000000029715 35945714 PMC9351907

[49] Altunay B Schäfer L Morgenroth A Peña Q Lammers T Saar M. Combining PSMA-targeted radiopharmaceutical therapy with immunotherapy. J Nucl Med. (2025) 66(10):1522–7. 10.2967/jnumed.125.270317 40876956

[50] Sandhu S Joshua AM Emmett L Spain LA Horvath L Crumbaker M. PRINCE: phase 1 trial of [^177^Lu]lu-PSMA-617 in combination with pembrolizumab in patients with metastatic castration-resistant prostate cancer (mCRPC). J Clin Oncol. (2022) 40(16_suppl):5017. 10.1200/JCO.2022.40.16_suppl.5017

[51] Müller C Umbricht CA Gracheva N Tschan VJ Pellegrini G Bernhardt P. [^161^tb]tb for PSMA-targeted radionuclide therapy of prostate cancer. Eur J Nucl Med Mol Imaging. (2019) 46(9):1919–30. 10.1007/s00259-019-04345-0 PMC6820371 31134301

[52] Larouze A Alcocer-Ávila M Morgat C Champion C Hindié E. Membrane and nuclear absorbed doses from [^177^Lu]lu and [^161^Tb]tb in tumor clusters: effect of cellular heterogeneity and potential benefit of dual targeting—a monte carlo study. J Nucl Med. (2023) 64(10):1619–24. 10.2967/jnumed.123.265509 37321819

[53] Buteau JP Kostos L Jackson PA Xie J Haskali MB Alipour R. First-in-human results of [^161^Tb]tb-PSMA-I&T dual beta-auger radioligand therapy in patients with metastatic castration-resistant prostate cancer (VIOLET): a single-centre, single-arm, phase 1–2 study. Lancet Oncol. (2025) 26(8):1009–17. 10.1016/S1470-2045(25)00332-8 40617237

[54] Schaefer-Schuler A Burgard C Blickle A Maus S Petrescu C Petto S. [^161^tb]tb-PSMA-617 radioligand therapy in patients with mCRPC: preliminary dosimetry results and intra-individual head-to-head comparison to [^177^Lu]lu-PSMA-617. Theranostics. (2024) 14(5):1829–40. 10.7150/thno.92273 38505615 PMC10945337

[55] Rosar F Burgard C Petrescu C Blickle A Bartholomä M Maus S. Pilot experience of [^161^Tb]tb-PSMA-617 RLT in mCRPC patients after conventional PSMA RLT within a prospective registry. Theranostics. (2025) 15(17):9019–28. 10.7150/thno.115831 40963912 PMC12439265

[56] Kurth J Krause BJ Schwarzenböck SM Bergner C Hakenberg OW Heuschkel M. First-in-human dosimetry of gastrin-releasing peptide receptor antagonist [^177^Lu]lu-RM2: a radiopharmaceutical for the treatment of metastatic castration-resistant prostate cancer. Eur J Nucl Med Mol Imaging. (2020) 47(1):123–35. 10.1007/s00259-019-04504-3 31482426

[57] Goodwin JF Schiewer MJ Dean JL Schrecengost RS De Leeuw R Han S. Androgen receptor signaling regulates DNA repair in prostate cancers. Cancer Discov. (2013) 3(11):1254–71. 10.1158/2159-8290.CD-13-0108 24027197 PMC3823813

[58] ClinicalTrials.gov. A phase Ib/II open-label, multi-center study of AMO959 with lutetium (^177^Lu) vipivotide tetraxetan (AAA617) in combination with an androgen receptor pathway inhibitor (ARPI) in adult participants with PSMA-positive metastatic castration-resistant prostate cancer (mCRPC). Identifier: NCT07226986. Available online at: https://clinicaltrials.gov/study/NCT07226986 (Accessed September 8, 2026).

[59] Mabikayi RM Kituku IM Kitoko R Komoy BA Bibelayi DD Kankolongo JN. In silico investigation of the interactions of piflufolastat F-18 and flotufolastat F-18 radiopharmaceuticals with biological targets relevant to prostate cancer diagnosis. Rev Sci Sant. (2026) 5(1):27–34. 10.71004/rss.026.v5.i1.50

